# Inflammation and cancer: friend or foe?

**DOI:** 10.3389/fphar.2024.1385479

**Published:** 2024-05-10

**Authors:** Andrés David Turizo-Smith, Samantha Córdoba-Hernandez, Lidy Vannessa Mejía-Guarnizo, Paula Stefany Monroy-Camacho, Josefa Antonia Rodríguez-García

**Affiliations:** ^1^ Doctorado en Oncología, Departamento de Patología, Facultad de Medicina, Universidad Nacional de Colombia, Bogotá, Colombia; ^2^ Semillero de Investigación en Cannabis y Derivados (SICAD), Universidad Nacional de Colombia, Bogotá, Colombia; ^3^ Facultad de Ciencias, Maestría en Ciencias, Microbiología, Universidad Nacional de Colombia, Bogotá, Colombia; ^4^ Grupo de investigación en Biología del Cáncer, Instituto Nacional de Cancerología, Bogotá, Colombia

**Keywords:** chronic inflammation, cancer, obesity, immune cells, cannabinoids, endocannabinoid system, non-steroidal anti-inflammatory drugs

## Abstract

Chronic inflammation plays a crucial role in the onset and progression of pathologies like neurodegenerative and cardiovascular diseases, diabetes, and cancer, since tumor development and chronic inflammation are linked, sharing common signaling pathways. At least 20% of breast and colorectal cancers are associated with chronic inflammation triggered by infections, irritants, or autoimmune diseases. Obesity, chronic inflammation, and cancer interconnection underscore the importance of population-based interventions in maintaining healthy body weight, to disrupt this axis. Given that the dietary inflammatory index is correlated with an increased risk of cancer, adopting an anti-inflammatory diet supplemented with nutraceuticals may be useful for cancer prevention. Natural products and their derivatives offer promising antitumor activity with favorable adverse effect profiles; however, the development of natural bioactive drugs is challenging due to their variability and complexity, requiring rigorous research processes. It has been shown that combining anti-inflammatory products, such as non-steroidal anti-inflammatory drugs (NSAIDs), corticosteroids, and statins, with plant-derived products demonstrate clinical utility as accessible adjuvants to traditional therapeutic approaches, with known safety profiles. Pharmacological approaches targeting multiple proteins involved in inflammation and cancer pathogenesis emerge as a particularly promising option. Given the systemic and multifactorial nature of inflammation, comprehensive strategies are essential for long term success in cancer therapy. To gain insights into carcinogenic phenomena and discover diagnostic or clinically relevant biomarkers, is pivotal to understand genetic variability, environmental exposure, dietary habits, and TME composition, to establish therapeutic approaches based on molecular and genetic analysis. Furthermore, the use of endocannabinoid, cannabinoid, and prostamide-type compounds as potential therapeutic targets or biomarkers requires further investigation. This review aims to elucidate the role of specific etiological agents and mediators contributing to persistent inflammatory reactions in tumor development. It explores potential therapeutic strategies for cancer treatment, emphasizing the urgent need for cost-effective approaches to address cancer-associated inflammation.

## 1 Introduction

Inflammation, particularly chronic inflammation, plays a pivotal role in the onset and progression of various chronic pathologies including neurodegenerative and cardiovascular diseases (Stephenson et al., 2018; Becatti et al., 2018), diabetes (Turkmen, 2017), and cancer (Murata, 2018). Breast and colorectal cancer (CRC) are the most prevalent types of cancer worldwide (Harbeck and Gnant, 2017), with a significant incidence and mortality rates (Sung et al., 2021). At least 20% of cancers are linked to chronic inflammatory processes triggered by factors such as infections, exposure to irritants, or autoimmune diseases (Table 1) (Crusz and Balkwill, 2015).

**TABLE 1:** Summary of risk factors and inflammatory conditions associated with cancer development and estimated new cases around the world. Globocan, 2020.

Type of cancer. International classification of diseases for oncology (ICD)	New estimated cases Globocan 2020	Inflammation related risk factors
Colorectal	1.931.590	Obesity, sedentary lifestyle
C18-21		tobacco use, alcohol consumption, inflammatory
		bowel disease
Breast cancer C50	2.261.419	Obesity, tobacco use, sedentary lifestyle, alcohol consumption
Lung C33-34	2.206.771	Tobacco use and exposure to environmental pollutants
Stomach C16	1.089.103	*H. Pylori* infection, obesity, alcohol consumption
Pancreatic C25	495.773	Tobacco use, chronic pancreatitis, diabetes, obesity
Liver	905.677	Hepatitis B and C virus
C22		infection, alcohol consumption, obesity
		diabetes, tobacco use
Leukemia C91-95	474.519	Obesity, tobacco use, human T-cell lymphotropic virus type 1 (HTLV-1) infection
Uterine cervix C54	604.127	Human papillomavirus (HPV) infection

Source: Adapted from (Todoric et al., 2016; Ferlay et al., 2021).

After successfully resolving the acute phase, the tissue remodeling process begins, aiming to reinstate physiological conditions. Inflammation, a physiological response, acts as a defense mechanism against pathogens and facilitates tissue repair once the triggering agent has been eliminated. Inflammatory response is dynamic and can manifest as an acute, self-limited process that effectively restores tissue homeostasis, by generating cellular determinants and locally active inflammatory mediator molecules (Gupta et al., 2018a).

Following the effective resolution of the acute phase, a subsequent stage of tissue remodeling is initiated to systematically restore the physiological condition of the affected area. However, the course of inflammation can deviate towards a chronic course when this regulatory mechanism is disrupted, impeding the resolution process. In such instances, the persistent production of proinflammatory molecules and the ongoing recruitment of inflammatory cells contribute to a chronic state, this prolonged inflammation hampers the re-establishment of tissue homeostasis, underscoring the complexity and potential consequences of dysregulated inflammatory responses (Sugimoto et al., 2016).

Tumor development is associated with a chronic inflammatory process, as inflammation and cancer both engage common signaling pathways (Barabutis et al., 2018). This review aims to elucidate specific etiological agents and mediators that can initiate persistent inflammatory reactions contributing to the development of tumors. Additionally, the exploration will encompass viable therapeutic strategies designed for cancer treatment, improving our understanding of the interplay between inflammation and tumorigenesis.

Presently, cancer treatments are prolonged and often involve several adverse effects. Moreover, their exorbitant costs render them inaccessible to more than 80% of the global population (Gupta et al., 2018a), Consequently, there is an imperative need to explore novel cost-effective therapeutic approaches for addressing the inflammatory phenomena associated with cancer.

## 2 Inflammation and cancer: a complex problem with no simple answers

It is now well-established that the presence of inflammatory cells precedes tumor development (Colotta et al., 2009; Thompson et al., 2015). Given that all tumors exhibit an inflammatory infiltration, chronic inflammation is widely recognized as a hallmark of cancer (Grivennikov et al., 2010; Hanahan and Weinberg, 2011; Capece et al., 2018; Paul, 2020). Carcinogenesis linked to factors such as tobacco use, pathogenic infections, and exposure to irritants like asbestos (Colotta et al., 2009; Thompson et al., 2015) explains the impact of inflammatory processes on tumor development. Inflammation promotes the acquisition of tumor cell characteristics such as apoptosis suppression, uncontrolled growth, tumor dissemination, and immune evasion (Colotta et al., 2009; Thompson et al., 2015). The inclusion of Inflammation as a distinctive feature of cancer stems from the pivotal role played by inflammatory cells in tumor development (Hanahan and Weinberg, 2011).

Through the action of proinflammatory agents such as histamine, growth factors, cytokines, and free radicals, among others, vascular permeability increases, creating a favorable environment for cancer development (Nagy et al., 2008). Cigarette smoking has been linked to a significant reduction in the activity of superoxide dismutase and glutathione peroxidase enzymes in erythrocytes, decreasing antioxidant defense mechanisms and increasing the cellular vulnerability to oxidative stress and damage associated with reactive oxygen species, potentially leading to carcinogenesis (Caliri et al., 2021).

The status of the human microbiome emerges as a risk factor influencing the development and outcome of various cancers (Aarnoutse et al., 2019; Lucas et al., 2017). It is well known that the human gastrointestinal system hosts approximately 1,000 species of microorganisms, which maintain symbiotic relationships with the host (Qin et al., 2010; Lucas et al., 2017), performing metabolic, immunological, and protective functions. An imbalance in its composition (dysbiosis) has been linked to chronic inflammatory diseases (Lucas et al., 2017). Numerous studies reveal alterations in the microbiome composition of patients with colon adenomas, suggesting a role for dysbiosis in the initial stages of CRC development (Shen et al., 2010; McCoy et al., 2013) and intestinal barrier dysfunction (Yu and Fang, 2015; Vipperla and Keefe, 2016).

Adopting healthy lifestyle including sound dietary consumption is fundamental for inflammation prevention, especially when coupled with factors such as obesity (Kolb et al., 2016) and cardiovascular disease (Becatti et al., 2018), which are closely associated with chronic diseases and cancer development (Escobar et al., 2020; Guha et al., 2021), It has been demonstrated that direct consumption of probiotics and/or prebiotics, like lactic acid bacteria, can modulate the development of certain types of cancer. Probiotics, defined as live microorganisms, conferring health benefits when administered in adequate amounts (Hill et al., 2014; Lucas et al., 2017; Cheng et al., 2020), have been reported to influence colorectal adenocarcinoma cell lines such as HT-29, leading to over-regulation of BAX and IL-10 expression and downregulation of Bcl-2 expression, inducing apoptosis, and inhibiting cell growth (Chen et al., 2017; Del Carmen et al., 2017). Furthermore, the microbiome’s composition influences responses to chemotherapy and immunotherapy (Iida et al., 2013; West and Powrie, 2015). In murine melanoma models, Bifidobacterium significantly improves the response to anti-PDL1 immunotherapy (Sivan et al., 2015), and *Bacteroides* enhances the effectiveness of anti-CTLA-4 therapy by stimulating the immune response (Vétizou et al., 2015).

Notably, inflammation observed in cancer patients demonstrates dysregulation and increased production of inflammatory agents like proteases, eicosanoids, cytokines, chemokines, and acute-phase inflammatory proteins that enter the circulation. Evidence suggests that immune cells produced many proteins, in broad quantities, regulating immune cells function under inflammatory conditions. Protein altered serum levels were observed in CRC patients compared to controls.

(Table 2) (Hanahan and Coussens, 2012; Candido and Hagemann, 2013; Tuomisto et al., 2019a). Evaluating the serum profiles of 13 cytokines, chemokines, and growth factors in 116 CRC patients and 86 healthy controls revealed an increased expression of IL-6, IL-7, CXCL8, IL-8, and PDGFB and decreased serum CCL2 levels (Kantola et al., 2012; Jin et al., 2014; Xu et al., 2016).

**TABLE 2:** Systemic inflammatory markers showing altered circulating levels in patients with CRC.

Acute-phase inflammatory proteins		
Marker	Function	Detection method
C-reactive protein (CRP) ↑	Acute-phase protein	ELISA (Enzyme Linked Immunosorbent Assays)
Ferritin ↓	Iron storage	Total Reflection X-Ray Fluorescence (TRXRF)
Cytokines and chemokines		
IL-6 ↑	Proinflammatory cytokine	ELISA
IL-7 ↑	Lymphocyte maturation	Magnetic microspheres
CCL2 ↓	Monocyte and macrophage recruitment	Multiplex magnetic microsphere assay
CXCL5 ↑	Neutrophil recruitment	ELISA
CXCL8 (IL8) ↑	Neutrophil recruitment	ELISA and Bio-Plex assay
CXCL10 ↑	T and NK cell recruitment	ELISA
Protease enzymes		
MMP9	Degradation of extracellular matrix and regulation of neutrophil action	ELISA
Growth factors		
PDGFB ↑	Mesenchymal cell proliferation	Multiplex magnetic microsphere assay
VEGFA ↑	Vascular endothelial growth factor	ELISA

Source: Adapted from (Tuomisto et al., 2019b).

Here, a concise overview is provided of some inflammatory cytokines and modulators that could serve as potential targets for anticancer therapy, along with their potential involvement in inflammation-associated carcinogenesis.

### 2.1 Interleukin-6 (IL-6)

IL-6, a pleiotropic cytokine, is produced by various immune cells (monocytes, macrophages, T and B lymphocytes), epithelial, fibroblast, glia, adipocytes, and tumor cells within the tumor microenvironment (TME) (Taniguchi and Karin, 2014a; Crusz and Balkwill, 2015). Notably, Ras-induced IL-6 secretion within the TME actively promotes tumor growth *in vivo* (Ancrile et al., 2007). IL-6 exhibits both pro- and anti-inflammatory actions contributing significantly to tumor development. Adipose tissue is a major source of IL-6, accounting for approximately 30% of circulating IL-6. This association explains the heightened cancer risk and poor prognosis associated with obesity (Kolb et al., 2016; Tuomisto et al., 2019a). Given that the clinical management practices for cancer patients are similar regardless of weight, understanding the mechanisms through which obesity influences cancer initiation and progression becomes crucial for developing precise therapies tailored to obese cancer patients.

In experimental models of carcinogenesis, IL-6 activity, mediated by signal transducer and activator of transcription 3 STAT3, increases cell survival, and promotes invasion and metastasis (Taniguchi and Karin, 2014a; Hirano, 2021a). It has been observed that an intriguing inverse relationship between IL-6 levels and response to treatments such as chemotherapy and hormone therapy. This correlation appears to align with a worse prognosis in various cancer types, including ovarian (Kulbe et al., 2007; Nolen et al., 2008; Anglesio et al., 2011; Kumari et al., 2016), hepatocellular (Naugler et al., 2007), and colorectal (Lippitz and Harris, 2016) cancers, among others. These insights underscore the significance of unraveling IL-6-mediated mechanisms in cancer progression and refining therapeutic strategies for improved patient outcomes.

### 2.2 Tumor necrosis factor α (TNFα)

TNFα exerts pleiotropic actions in the regulation of the inflammatory immune response. The interaction of TNFα with both TNFR1 and TNFR2 receptors governs the modulation of cytokines, proteases, and growth factors (Crusz and Balkwill, 2015). In chemically induced models of colitis and CRC, TNFα produced by mononuclear cells appears to play a pivotal role in inflammation and subsequent tumor development (Popivanova et al., 2008). Therapeutic interventions involving anti-TNFα antibodies or TNF receptor fusion molecules have demonstrated efficacy in genetic models of liver cancer and CRC, although the precise mechanism of action of these therapies remains to be elucidated (Pikarsky et al., 2004; Rao et al., 2006).

### 2.3 NF-kB

NF-kB, a key regulator of inflammatory events, is associated with tumor development and progression (Capece et al., 2018). This transcription factor orchestrates inflammatory immune responses and governs various aspects of tumor development, including inhibition of apoptosis, stimulation of cell proliferation, and promotion of cell migration and invasion (Ben-Neriah and Karin, 2011; DiDonato et al., 2012). In CRC, NF-kB hampers the efficacy of chemotherapeutic agents such as 5-fluorouracil, oxaliplatin, and paclitaxel by upregulating the expression of anti-apoptotic proteins, like Bcl-2 and Bcl-xL (Soleimani et al., 2020), Furthermore, NF-kB promotes the expression of proinflammatory cytokines, including TNFα, IL-6, and IL-1β, elevates levels of angiogenic factors such as HIF-1α, IL-8, and vascular endothelial growth factors (VEGF), and facilitates the expression of chemokines, cytoskeleton genes, and matrix metalloprotease (MMP) (Patel et al., 2018; Martin et al., 2021) all contributing to a microenvironment favorable for metastasis.

### 2.4 Signal transducer and activator of transcription 3 (STAT3)

STAT3 emerges as a pivotal transcription factor involved in cancer progression, with its persistent activation fostering chronic inflammation that increases cellular susceptibility to carcinogenesis. STAT3 promotes signaling through pro-oncogenic inflammatory pathways, such as NF-kB and gp130/Jak/STAT, leading to increased tumor cell proliferation, survival, and invasion while simultaneously suppressing antitumor immunity (Yu et al., 2009; Loh et al., 2019). STAT3 plays a crucial role in the carcinogenesis and tumor progression of various solid tumors, including head and neck squamous cell carcinoma (Loh et al., 2019) and CRC (Gargalionis et al., 2021), as well as leukemias and lymphomas (Loh et al., 2019).

### 2.5 Cyclooxygenases (COX)

COX are enzymes with tumor-promoting activity. They achieve this by converting free arachidonic acid (AA) into prostanoids, including prostaglandins (PGs) (Wang and Dubois, 2010), which act on tumor cells by inhibiting apoptosis, increasing cell migration, and promoting angiogenesis (Crusz and Balkwill, 2015; Chatterjee et al., 2018). The continued overexpression of COX-2 plays a significant role in promoting carcinogenesis. It does so by increasing the expression of carcinogenic reactive oxygen species (ROS) and the production of prostaglandin E2 (PGE2). COX-2 further stimulates VEGF through PGE2 promoting angiogenesis and increasing the production of metalloproteinases to favor invasion and metastasis. Additionally, it decreases bioavailable arachidonic acid stores, reducing cell differentiation and apoptosis. Notably, COX-2 inhibits the proliferation of B and T lymphocytes, as well as NK cells, thereby limiting the antineoplastic activity of the immune system (Desai et al., 2018). COX-2 overexpression has been observed in various cancers, including breast, colon, prostate, pancreatic, head and neck, skin, and lung (Crusz and Balkwill, 2015; Chatterjee et al., 2018; Desai et al., 2018).

Experimental evidence suggests that COX can metabolize endocannabinoids, such as 2- Arachidonoylglycerol (2-AG) and Anandamide (AEA), into prostaglandin glycerol esters (PG-Gs) and prostaglandin ethanol amides (PG-EAs), respectively (Kozak et al., 2002). COX-2-derived metabolites, collectively known as prostamide, represent a novel class of biologically active eicosanoids. Prostamide modulates cellular functions including the modulation of IL-2 expression in T cells (Rockwell et al., 2008), the inhibition of cell growth, and the induction of apoptosis in CRC cell lines HT29 and HCA7/C29 (Patsos et al., 2005).

In contrast to prostaglandins, which are a well-established potent bioactive lipid messengers derived from arachidonic acid, with extensively studied physiological functions and receptor signaling pathways, the roles of prostamides remain not yet fully understood. Evidence suggests that uridine diphosphate (UDP) P2Y6 serves as the specific receptor for PGE2-G (Brüser et al., 2017). P2Y6 receptor, which under normal physiological conditions is expressed in various cell types and tissues, including the spleen, thymus, intestine, aorta, and leukocytes, plays a crucial role in maintaining immune functions. It has been shown that P2Y6 potentiates proinflammatory responses in macrophages and exhibits differential roles for the development of atherosclerotic lesions, as P2Y6 deficiency can reduce macrophage-mediated cholesterol uptake (Garcia et al., 2014).

Furthermore, the P2Y6 receptor serves as a significant endogenous inhibitor of T-cell function in allergic pulmonary inflammation (Giannattasio et al., 2011). P2Y6 is implicated in increasing IL-1β production and hyperalgesia, in inflammation and macrophage activation (Brüser et al., 2017). While classical PGE2 can reduce the production of proinflammatory cytokines after lipopolysaccharide (LPS) stimulation, limited information exists regarding the ability of prostamide, prostaglandin E2 ethanolamine (PGE2-EA), to modulate immune responses. However, it has been indicated that PGE2-EA, at low concentrations, is pharmacologically active in various smooth muscle tissues and can bind to all four PGE receptor subtypes (EP1-EP4), suggesting similar actions to PGE2. Additionally, studies have shown that PGE2-EA can suppress the inflammatory action of human monocytes by inhibiting LPS- stimulated TNF-α production (Brown et al., 2013).

While numerous studies demonstrate the production of PGE2 at inflammatory sites, immunoassays quantifying PG have indicated non-specific binding to structurally similar compounds such as PGE2-EA (Glass et al., 2005). Chromatographic methods are recommended for accurate analyses, as prostamides were initially misidentified as prostanoids (Woodward et al., 2008; Brown et al., 2013; Gouveia-Figueira and Nording, 2015). Notably, prostamides exhibit a longer half-life compared to prostaglandins. Even though the latter have substantial biological effects. Prostamide and glycerol esters as endocannabinoid-derived COX-2 metabolites may be stable enough to exert systemic activity (Weber et al., 2004).

Building up the information provided above, prostamide-type compounds emerge as potential biomarkers and therapeutic targets for addressing inflammation and influencing adipocyte differentiation in a coordinated effort to regulate carcinogenic processes. Notably, there is a perspective among some authors suggesting that the potential therapeutic advantage of COX-2 inhibitors may, at least in part, stem from their ability to diminish or modulate prostamide levels (Woodward et al., 2008). This insight underlines the potential significance of targeting prostamide pathways in the development of therapeutic interventions for conditions involving inflammation and adipocyte differentiation, contributing to the overall orchestration of carcinogenic phenomena.

## 3 Immune cells associated with tumor microenvironment

**FIGURE 1:**
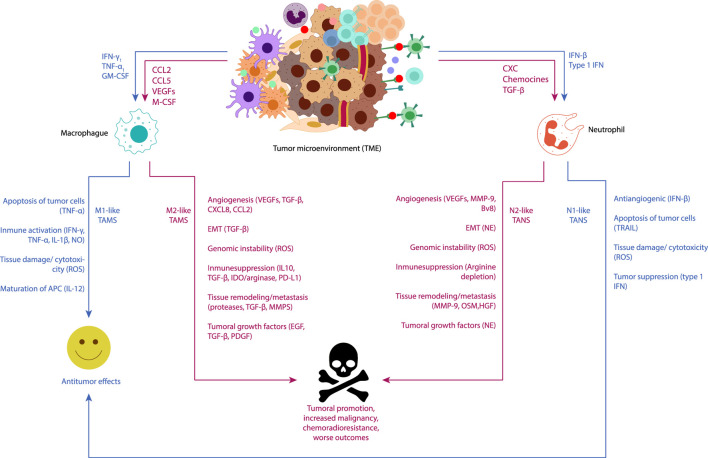
Visual representation of the functions of tumor-associated macrophages (TAMs) and tumor-associated neutrophils (TANs) in cancer-related inflammation underscores their pivotal roles as key regulators of tumor-related inflammatory processes. Neoplastic and stromal cells recruit macrophages and neutrophils to the tumor microenvironment (TME), directing their polarization towards different phenotypes. Macrophages can polarize into an M1-like phenotype with antitumor functions under immunostimulatory cytokines such as IFN-γ and TNF-α, releasing TNF-alpha, ROS, and NO to induce apoptosis and cytotoxicity in cancer cells. Conversely, M2-like macrophages, conditioned by the hypoxic tumor microenvironment and immunosuppressive mediators (IL-10, TGFβ), exhibit pro-tumor functions. M2-like TAMs secrete molecules promoting angiogenesis (CXCL8, VEGF), tumor proliferation (EGF, TGF-β, PDGF), induce epithelial-mesenchymal-transition (EMT) (TGFβ), and continuous matrix remodeling (MMPs, proteases). They also produce various immunosuppressive molecules (IL-10, TGFβ, IDO1/2), which support regulatory T cells. Neutrophils, under TGF-β induction, can polarize into N1 phenotype, whereas under the influence of type I IFNs, they polarize into N2 phenotype. Subsequently, N1 neutrophils could inhibit the development of cancer through tumor cell cytotoxicity, tumor suppression (type 1 IFN), and antiangiogenic effects over the tumor (IFN-β). On the other hand, N2 neutrophils could promote the development of cancer by fostering carcinogenesis and cancer metastasis [MMPs, oncostatin M (OSM), hepatocyte growth factor (HGF)], tumor growth (NE), and cancer angiogenesis [VEGFs, MMP-1, prokineticin 2 (BV8)], as well as suppressing immunity (arginine depletion).

Immune cells play a pivotal role in maintaining tissue homeostasis and eliminating pathogens or damaged cells. However, in the TME, the dynamics of immune recognition and cytotoxicity are altered, favoring cell survival, and facilitating tumor development (Figure 1) (Khandia and Munjal, 2020).

Tumor-associated macrophages (TAMs), which represent up to 50% of the tumor mass within the TME, play a significant role in promoting cell proliferation, suppressing the antitumor immune response, and enhancing immune evasion and metastasis. Recruitment of TAMs to the TME from the bloodstream is orchestrated by the action of cytokines, chemokines, and growth factors produced by tumor and stromal cells (Todoric et al., 2016). Typically, macrophages recruited to the TME undergo reprograming from an antitumor M1 phenotype to a pro tumor M2 phenotype, which promotes tumor development (Todoric et al., 2016; Khandia and Munjal, 2020). In various cancer types, the infiltration of M2 macrophages has been linked to a poor prognosis due to their tumor growth-promoting functions. This association has stimulated the exploration of novel therapeutic alternatives aimed at reducing the infiltration of these cells in the microenvironment of different tumors both *in vivo* and *in vitro*.

Similar to macrophages, neutrophils also play crucial roles in modulating tumor behavior, as evidenced by experimental models and epidemiological studies. Tumor-Associated Neutrophils (TANs) are key components in Cancer-Related Inflammation, exhibiting versatile functions that can either impede or promote tumor progression. In murine cancer models, neutrophils have been shown to respond to TGF-β by acquiring a protumoral phenotype. However, inhibition of TGF-β leads to enhanced neutrophil infiltration into tumors, resulting in heightened cytotoxicity against tumor cells and elevated expression of pro-inflammatory molecules. Additionally, neutrophils possess a spectrum of activation states, including an antitumor N1 phenotype and a protumor N2 phenotype, in response to signals from the Tumor Microenvironment (TME). These findings underscore the complexity of immune cell interactions within the TME and highlight the potential of targeting neutrophils as a therapeutic strategy in cancer treatment (Galdiero et al., 2018).

Numerous research studies have accelerated the development of potential therapies, including the use of antibodies like anti-colony-stimulating factor receptor 1 (CSF-1R), which has shown clinical benefits for patients with diffuse multinucleated giant cell tumors (Mantovani and Allavena, 2015). Moreover, considering that TAMs express substantial amounts of COX-2, experiments in animal models of colon cancer, have demonstrated that the use of COX-2 inhibitors such as celecoxib, results in a phenotypic shift from M2 to M1, resulting in polyp reduction (Nakanishi et al., 2011), highlighting promising avenues for therapeutic interventions targeting immune cells within the tumor microenvironment.

**FIGURE 2:**
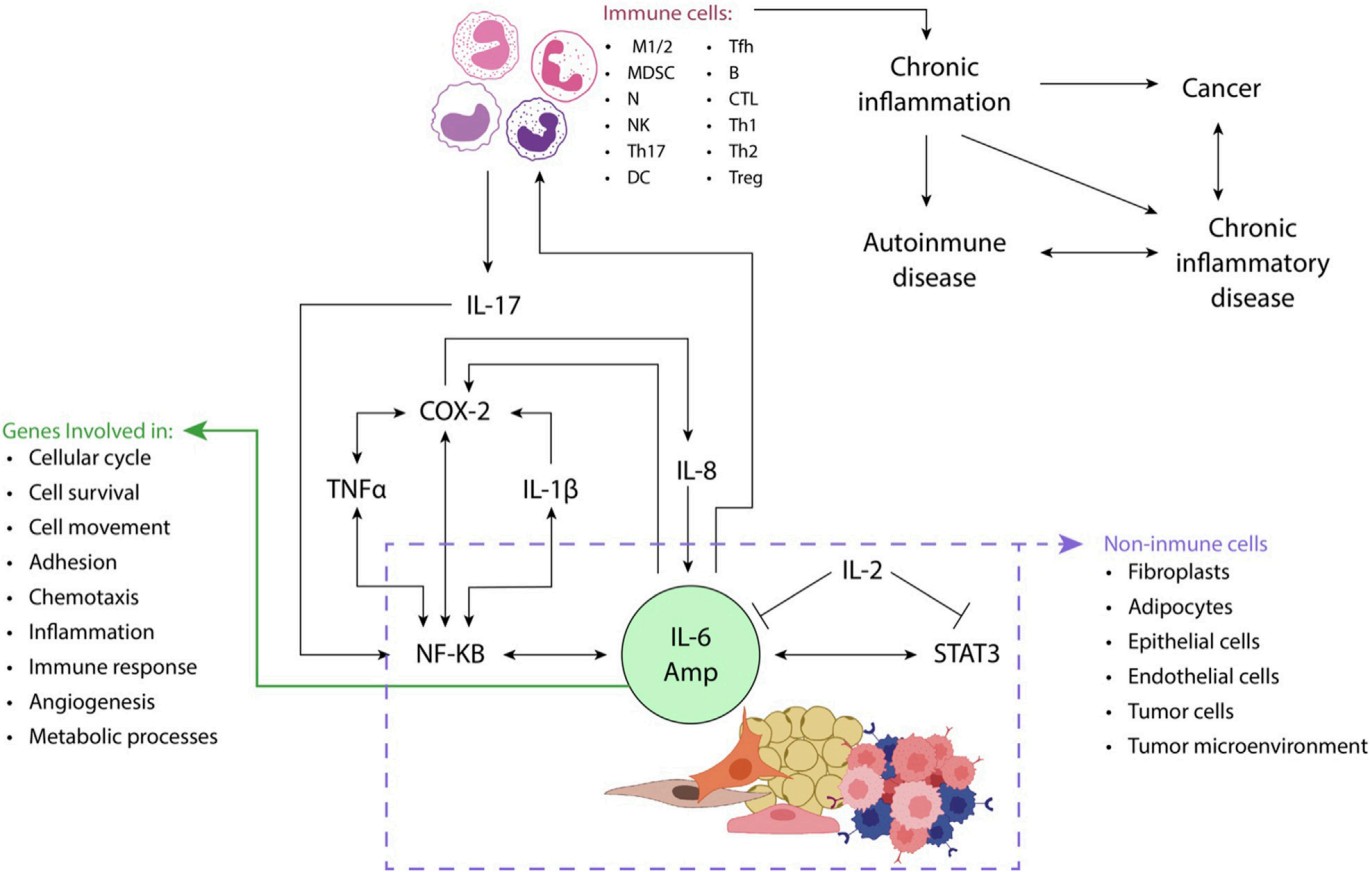
STAT3 (e.g., IL-6) and NF-κB (e.g., IL-17 or TNFα) activators play a crucial role in enhancing the production of proinflammatory mediators, including IL-6, from non-immune cells such as fibroblasts and adipocytes. This process gives rise to an amplification mechanism known as the IL-6 amplifier (IL-6 Amp). Through the production of chemokines, IL-6 Amp facilitates the recruitment of immune system cells such as activated T-cells to the site of inflammation. The dynamic interaction between immune and non-immune cells further reinforces the IL-6 Amp, leading to an increased expression of genes involved in diverse cellular processes, including cell proliferation, survival, and migration, inflammation, and angiogenesis, among others. This intricate interplay underscores the significance of STAT3 and NF-κB activation in orchestrating a cascade of events that contribute to the sustained and heightened inflammatory response within the affected tissue implicated in cancer and chronic inflammatory diseases. Adapted from (Hirano, 2021b).

**FIGURE 3:**
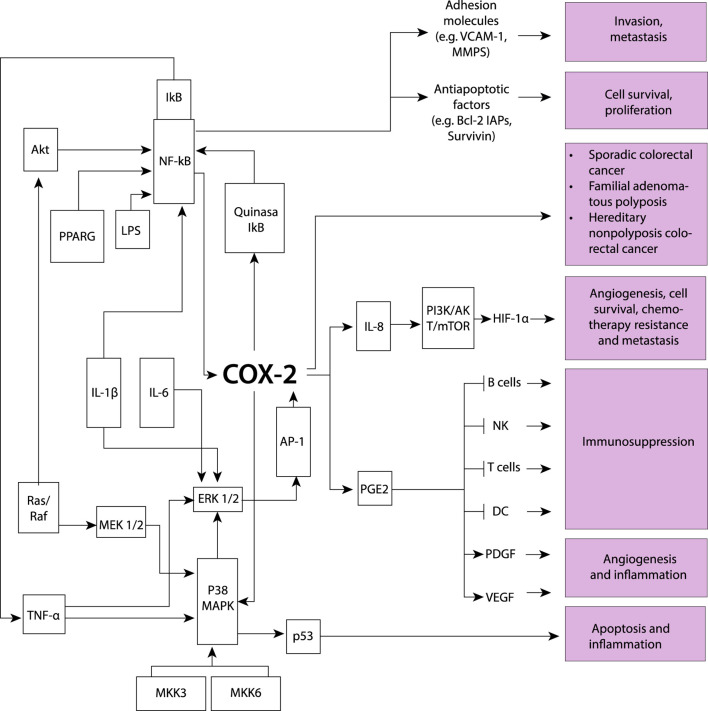
The induction of the COX-2 model involves various key processes driven by proinflammatory cytokines, resulting in stimulated cell proliferation and increased metastatic potential, while also influencing apoptosis and inflammation. Several molecular components participate in this intricate network: Akt protein kinase B (AKT-PKB), involved in signaling pathways related to cell survival and growth; activator protein-1 (AP-1), pivotal in cellular processes such as proliferation, differentiation, and apoptosis; cyclooxygenase-2 (COX-2), playing a role in inflammation and promoting cell proliferation; extracellular signal-regulated kinases 1/2 (ERK1/2), contributing to cell proliferation and differentiation; inhibitor of κB (IκB), regulating inflammatory responses; interleukins (IL), involved in immune responses and inflammation; lipopolysaccharide (LPS), a component of Gram-negative bacterial cell walls inducing inflammation; mitogen-activated protein kinase 1/2 (MEK1/2), playing a role in cell proliferation; mitogen-activated protein kinase 3 (MKK3), contributing to cellular responses; nuclear factor-κB (NF-κB), central to the regulation of immune and inflammatory responses; P38 mitogen-activated protein kinase (P38 MAPK), involved in cellular responses to stress and inflammation; p53 oncogene (p53), regulating cell cycle progression and apoptosis; prostaglandin E2 (PGE2) with various physiological effects, including inflammation; peroxisome proliferator-activated receptor γ (PPARG), involved in regulating genes associated with adipocyte differentiation and inflammation; mitogen-activated protein kinase cascade (Ras/Raf), involved in cell growth and differentiation; tumor necrosis factor-α (TNF-α), involved in inflammation and immune system regulation; Phosphoinositide 3-kinase, Protein kinase B, and Mammalian target of rapamycin (PI3K/AKT/mTOR), regulate cell growth and survival; Hypoxia-inducible factor 1α (HIF-1α), controls the cellular response to low oxygen levels; Vascular endothelial growth factor (VEGF), promotes the formation of new blood vessels; Platelet-derived growth factor (PDGF), stimulates cell growth and angiogenesis; Dendritic cells (DC), serve as antigen-presenting cells; T lymphocytes (T cells), coordinate specific immune responses against pathogens; Natural killer cells (NK), are immune cells that target and eliminate infected or cancerous cells; B lymphocytes (B cells), produce antibodies as part of the immune response; B-cell lymphoma 2 (BCL-2), is a protein involved in regulating apoptosis or programmed cell death; Inhibitor of apoptosis proteins (IAPs), regulate cell death processes; Survivin is a protein that inhibits apoptosis and promotes cell survival; Vascular cell adhesion molecule 1 (VCAM-1), is involved in inflammation and immune cell adhesion; Matrix metalloproteinases (MMPs), are enzymes responsible for degrading extracellular matrix components contributing to tissue remodeling and invasion. COX-2 is also associated with sporadic colorectal cancer development, as well as in familial adenomatous polyposis and hereditary colorectal cancer, indicating its involvement across different forms of diseases.

Figures 2, 3 illustrate the interactions between some inflammatory regulatory agents and their role in carcinogenesis.

## 4 Obesity, inflammation, and cancer

Obesity is an increasing public health problem impacting 35% of US adults and escalating the risk of numerous cancer types, often correlating with unfavorable outcomes. Central to the progression of chronic conditions is chronic inflammation, characterized by an obesity-associated phenotype (Kolb et al., 2016). This inflammatory response initiates with an excessive nutrient intake and manifests in specialized metabolic tissues. White adipose tissue adipocytes are endocrine cells that secrete a spectrum of cytokines, hormones, and growth factors playing a pivotal role in obesity-associated inflammation (Gregor and Hotamisligil, 2011; Fasshauer and Blüher, 2015). Dysregulation of metabolic signaling pathways, including NF-κB, c-Jun N-terminal kinase (JNK), nuclear factor B, and protein kinase R results from unhealthy lifestyles and imbalanced dietary habits (Kolb et al., 2016).

Simultaneously, obesity triggers increased endoplasmic reticulum (ER) stress, activating of NF- κB and JNK, escalating oxidative stress, and positively regulating proinflammatory cytokines (Cnop et al., 2012). All these pathways collectively contribute to the initiation of obesity-associated inflammation. Although localized in white adipose tissue, this low-grade inflammatory response extends its influence on other tissues like the liver, pancreas, and brain (Cildir et al., 2013). Furthermore, the inflammatory response linked to obesity leads to changes in immune cell infiltration and polarization within obese white adipose tissue compared with lean white adipose tissue (Han and Levings, 2013).

Macrophages, the primary immune cell population recruited to white adipose tissue serve as a significant source of proinflammatory cytokines in this context (Weisberg et al., 2003). The adipose tissue of obese individuals not only features increased macrophages but also tends to shift them from an anti-inflammatory M2 phenotype to a proinflammatory M1 phenotype (Lumeng et al., 2007), partly due to an imbalance of obesity-related adipokines (e.g., leptin to adiponectin ratio). This dysregulation, marked by elevated leptin production (proinflammatory, pro-angiogenic, and pro-proliferative) and reduced adiponectin production (anti-inflammatory, anti-angiogenic, and anti-proliferative) (Kolb et al., 2016), is associated with an increased risk of colon cancer in men due to the association with high leptin levels (Stattin et al., 2003; Stocks et al., 2008). Leptin has been demonstrated to promote proliferation, survival, and invasive potential in colon cancer cells by activating MAPK, PI3K, NF-κB, and STAT3 signaling (Rouet-Benzineb et al., 2004; Uchiyama et al., 2011; Wang et al., 2012).

Studies suggest that white adipose tissue macrophages in obese individuals express inflammatory cytokines associated with M1 macrophages but lack other M1 macrophage characteristics (Kratz et al., 2014). These macrophages along with adipocytes, release over 50 different cytokines, hormones, and chemokines, contributing to the chronic inflammation associated with obesity (Balistreri et al., 2010; Sun et al., 2011). Inflammation and inflammatory cytokines play a pivotal role in the development of colitis-associated sporadic CRC (Terzic et al., 2010; Moossavi and Bishehsari, 2012). Altered expression of TNF-α, a potent inducer of IL-6, has been observed to promote the development of colorectal and breast cancer (Sun et al., 2012; Taniguchi and Karin, 2014b). Moreover, a plausible link between obesity and increased incidence of Estrogen Receptor-positive (ER+) breast cancer in postmenopausal women has been noted, due to elevated circulating estrogen levels resulting from increased androgen aromatization in adipose tissue (Lorincz and Sukumar, 2006; Kolb et al., 2016).

## 5 Senescence

Age stands out as the most significant risk factor for cancer development as evidenced by an exponential increase in the incidence of most cancers with advancing age (Hsu, 2016). This observation suggests the existence of aging-associated factors that contribute to the initiation and progression of tumors. Certain features of aging, including epigenetic changes, mitochondrial dysfunction, and alterations in proteostasis, among others, share commonalities with characteristics observed in carcinogenesis. Contrary to the initial belief that senescent cells arrested tumor progression due in part to their characteristic growth arrest, accumulating evidence indicates that these cells present in higher numbers in older individuals and cancer patients undergoing chemotherapeutic treatment, remain metabolically active. Moreover, they secrete a combination of inflammatory agents, growth factors, and proteases, collectively referred to as senescence-associated secretory phenotype (SASP) (López-Otın et al., 2013). SASP manifests as an inflammatory dysregulation observed in aged mammals and is associated with tumor initiation and progression. It contributes to the creation of a favorable TME or directly influences the intrinsic properties of tumor cells (Morales-Valencia and David, 2021). This revelation underscores the intricate relationship between aging, senescence, and cancer development, highlighting the dynamic and multifaceted nature of these processes.

Several studies have explored the impact of senescence on cancer initiation. In cellular models of lung (H460), colorectal (HCT116), and murine breast cancers (4T1), induced senescence by exposure to etoposide or doxorubicin, resulted in the renewal proliferation and the formation of tumors when implanted in immunodeficient or immunocompetent mice (Saleh et al., 2019). B-cell lymphoma models revealed that cells induced to senesce by doxorubicin exposure exhibited a more aggressive growth potential compared to non-senescent cells after chemotherapy (Milanovic et al., 2018). Moreover, the accumulation of senescent cells in adipose tissue, particularly in visceral fat, has been linked to obesity with obese individuals having up to 30 times more senescent cells than non-obese individuals (Tchkonia et al., 2010; Liu et al., 2019). The presence of proinflammatory senescent cells in obese individuals holds clinical implications, especially given the substantial size of adipose tissue. This is particularly relevant as obesity-associated inflammation is known to be linked to cancer incidence and progression. Obese patients express elevated levels of SASP components such as IL-6, IL-8, and TNF-α, reflecting the increased levels observed in older adults (Morales-Valencia and David, 2021).

**FIGURE 4:**
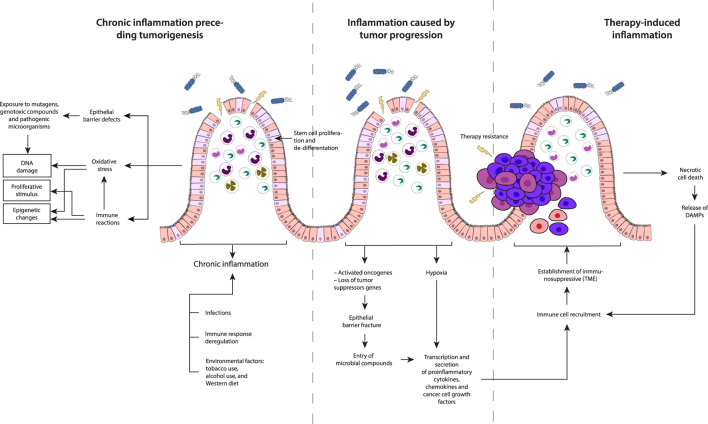
Description of the three types of inflammation based on their timing concerning cancer development: Chronic inflammation preceding tumorigenesis, Inflammation caused by tumor progression and Therapy-induced inflammation.

Forms of inflammation in carcinogenic development can be categorized based on their timing in cancer pathogenesis: 1) chronic inflammation preceding tumorigenesis, 2) inflammation caused by tumor progression, and 3) therapy-induced inflammation (Figure 4). Tumor cells interact with their microenvironment, comprising immune and stromal cells to suppress immune responses, while inflammatory processes shape the immune pathogenesis of cancer.

### 5.1 Chronic inflammation preceding tumorigenesis

It occurs before the initiation of tumorigenesis and is driven by factors such as infections, dysregulated immune responses, and environmental triggers. Environmental factors including Oxidative stress, exposure to mutagenic compounds, constant exposure to proliferative inflammatory stimuli, and defects in the epithelial barrier can initiate and promote chronic inflammatory reactions. The chronic inflammatory state induced by these factors may lead to DNA damage or epigenetic changes, creating a favorable environment for the development of tumors. Two events are crucial for inducing tumor development from normal cells: a tumorigenic event involving the accumulation of mutations or epigenetic alterations, and the clonal expansion of altered cells leading to tumor formation. Inflammation contributes to both events by inducing DNA damage, increasing oxidative stress from tissue-resident cells, or innate immune cells recruited to the TME in the absence of exogenous carcinogens (Meira et al., 2008; Schmitt and Greten, 2021).

### 5.2 Inflammation caused by tumor progression

This type of inflammation that supports tumor growth, is highly dependent on cell-cell interactions within the TME of sporadic tumors, promoting local growth and metastasis formation (Greten and Grivennikov, 2019). This protumor inflammatory response can be triggered by events such as hypoxia-induced cell death, fracture of the epithelial barrier and the subsequent entry of microbial compounds. These conditions within the tumor microenvironment contribute to the establishment of an inflammatory milieu that aids in the progression and survival of cancer cells. In CRC, loss of p53 function affects epithelial integrity activating NF-kB and STAT3 inflammatory pathways (Schwitalla et al., 2013). Hypoxia, nutrients deficiency in poorly vascularized tumor areas and necrotic cell death, lead to the secretion of proinflammatory factors (Jorgensen et al., 2017; Kim et al., 2019). Hypoxia also induces hypoxia-inducible factor 1α (HIF1α) expression, activating tumor-associated fibroblasts and recruiting immune cells, including monocytes, macrophages, and B-cells to the TME (Ammirante et al., 2014). In CRC, hypoxic stress, and elevated TGFβ levels, favor the differentiation into regulatory T-cells (Treg cells) suppressing effector T-cell differentiation and affecting antitumor immunity (Westendorf et al., 2017; Schmitt and Greten, 2021).

### 5.3 Therapy-induced inflammation

This is an unintended consequence of therapy rather than its objective; but it plays a decisive role in determining therapeutic response and the probability of relapse, exhibiting potential antitumor or protumor effects, depending on the specific context. Following therapy, tumor cells, undergo necrosis, releasing damage-associated molecules, or neoantigens, which recruit and activate antigen presenting cells (APCs) (Schmitt and Greten, 2021) which are capable of presenting neoantigens to T-cells and activating *de novo* responses and enhance immunosurveillance (Ghiringhelli and Fumet, 2019). However, this process can also contribute to tumorigenesis and the suppression of antitumor immunity. For instance, IL-1α released by necrotic cells promotes malignant transformation, angiogenesis, and metastasis. Simultaneously, it activates and polarizes fibroblasts towards an inflammatory phenotype, fostering tumorigenesis (Sahai et al., 2020). Conversely, ionizing radiation has been observed to increase the population of Treg cells in skin cancer (Price et al., 2015), while drugs like oxaliplatin induce the recruitment of plasmacytes expressing immunosuppressive markers such as IgA, IL-10, and PD-L1, in a TGF-β receptor signaling-dependent manner in prostate cancer (Shalapour et al., 2015). It is crucial to note that therapy-induced tumor cell death promotes the production of growth factors and cytokines, including WNT, EGF, TNF, IL-17, and IL-6, by cells in the TME to promote the survival of remaining tumor cells and play a role in fostering therapy resistance (Greten and Grivennikov, 2019). The release of damage-associated molecular patterns (DAMPs) from necrotic cells and the TME can trigger tumor-promoting inflammation that can impact the overall dynamics of the tumor microenvironment, influencing the course of cancer progression.

Understanding these distinct types of inflammation and their temporal association with cancer pathogenesis is crucial for developing targeted interventions and therapeutic strategies tailored to the specific inflammatory context at different stages of cancer development.

## 6 Therapeutic approaches in cancer

Therapeutic strategies targeting cancer-associated inflammation include the exploration of anti-inflammatory drugs, particularly non-steroidal anti-inflammatory drugs (NSAIDs), which function by inhibiting COX enzymes. Epidemiological and preclinical data indicate the potential utility of these drugs in cancer prevention and treatment (Crusz and Balkwill, 2015; Wong, 2019; Zappavigna et al., 2020; Michels et al., 2021). As an example, aspirin, an NSAID, has demonstrated promise in reducing the risk of esophageal, liver, breast, and colorectal cancer and it is associated with a reduced risk of metastasis, particularly in patients with adenocarcinomas (Table 3) (Algra and Rothwell, 2012; Rothwell et al., 2012).

**TABLE 3:** Preventive and anticancer effects of some anti-inflammatory drugs.

Medication	Effect
Aspirin	Preventive effect in breast, bladder, esophagus, lung, and colorectal cancers
Dexamethasone	In multiple myeloma, it induces cell death by expression of miR-125b. Preventive effect in breast cancer, multiple myeloma, and CRC
Ibuprofen	It inhibits β-catenin nuclear activation in human colon adenoma
Sulindac	It induces NF-κB pathway activation in colon cancer cells. Preventive effect on breast cancer
Statins	Preventive effect in renal cell carcinoma, lung, prostate, and colorectal cancers

Source: Elaborated from information about (Xiao and Yang, 2008; Zappavigna et al., 2020).

Despite these promising findings, it is crucial to exercise caution in the use of NSAIDs other than aspirin due to potential risks. The use of such drugs has been associated with an increased risk of bleeding, myocardial infarction, gastrointestinal bleeding, and renal failure. These adverse effects arise from the modulation of signaling pathways by NSAIDs, emphasizing the need for careful evaluation when considering the use of alternative NSAIDs for cancer treatment and prevention. It is essential to recognize that the underlying mechanisms and potential side effects of these drugs are not yet fully understood (Wong, 2019; Michels et al., 2021).

Another therapeutic approach for cancer-associated inflammation involves selectively suppressing crucial proinflammatory mediators, using blockers specifically targeting TNF-α (Hou et al., 2021), NF-κB (Shishodia et al., 2003; Capece et al., 2018), and COX-2 (Chatterjee et al., 2018). However, many potential inflammatory biomarkers lack cancer specificity (Eugen-Olsen et al., 2010), necessitating a thorough assessment to ensure their utility in patients follow-up, therapeutic response, and cancer-associated inflammation monitoring.

While C-reactive protein (CRP) has been suggested as a potential inflammation biomarker, its expression levels can be influenced by several factors. Additionally, the presence of multiple CRP isoforms makes it difficult to establish a direct relationship with cancer development (Roe, 2021a). Other markers, such as cytokines IL-6 (Masjedi et al., 2021), TNFα (Michels et al., 2021), and IL-8 (Khandia and Munjal, 2020), among others (Table 4), highlight the importance of establishing a classification system. Despite progress, there is currently no consensus on the causative factors that influence the levels of these markers in the context of cancer-associated inflammation (Roe, 2021a). Additional research is imperative to refine our understanding of the specificity of inflammatory markers (Bhavsar et al., 2014) while there is evidence indicating a pivotal role of proinflammatory cytokines, enzymes, and transcription factors in promoting tumorigenesis, it is important to note that the inhibition of proinflammatory pathways may not always be beneficial (Sethi et al., 2012). For instance, the transcription factor NF-kB, closely related to various cancer features (Capece et al., 2018), has been found to exert inhibitory effects on tumor development (Dajee et al., 2003). This duality underscores the complexity of inflammatory pathways, and inhibiting them might act as a double-edged sword, influencing tumor progression in multifaceted ways. Thus, understanding the intricate interplay between inflammation and cancer is essential to develop targeted and effective therapeutic interventions (Michels et al., 2021).

**TABLE 4:** Example of classification of inflammatory factors and effects on cytokine levels.

Inflammation type	Factor	IL-1β	TNF-α	IL-6	IL-8	IL-12	INF-α	IFN-γ
	Ischemic stroke	E	E	E				
	Atherosclerosis	E	E					E
VII	Infarction	E	E	E				
VI	Diabetes	E	E	E	E	E		E
	Osteoarthritis	E	E	E	E			
	Rheumatoid arthritis	E	E	E		E		
V	Colon cancer	E	E	E	E		E	
	Lung cancer		E	E	E			E
	Gastric cancer	E	E	E	E			
	Breast cancer	E	E	E	E			
	Esophageal cancer	E	E	E	E	E		D

Source: Adapted from (Roe, 2021b). E, Elevated; D, Decreased below normal levels.

**FIGURE 5:**
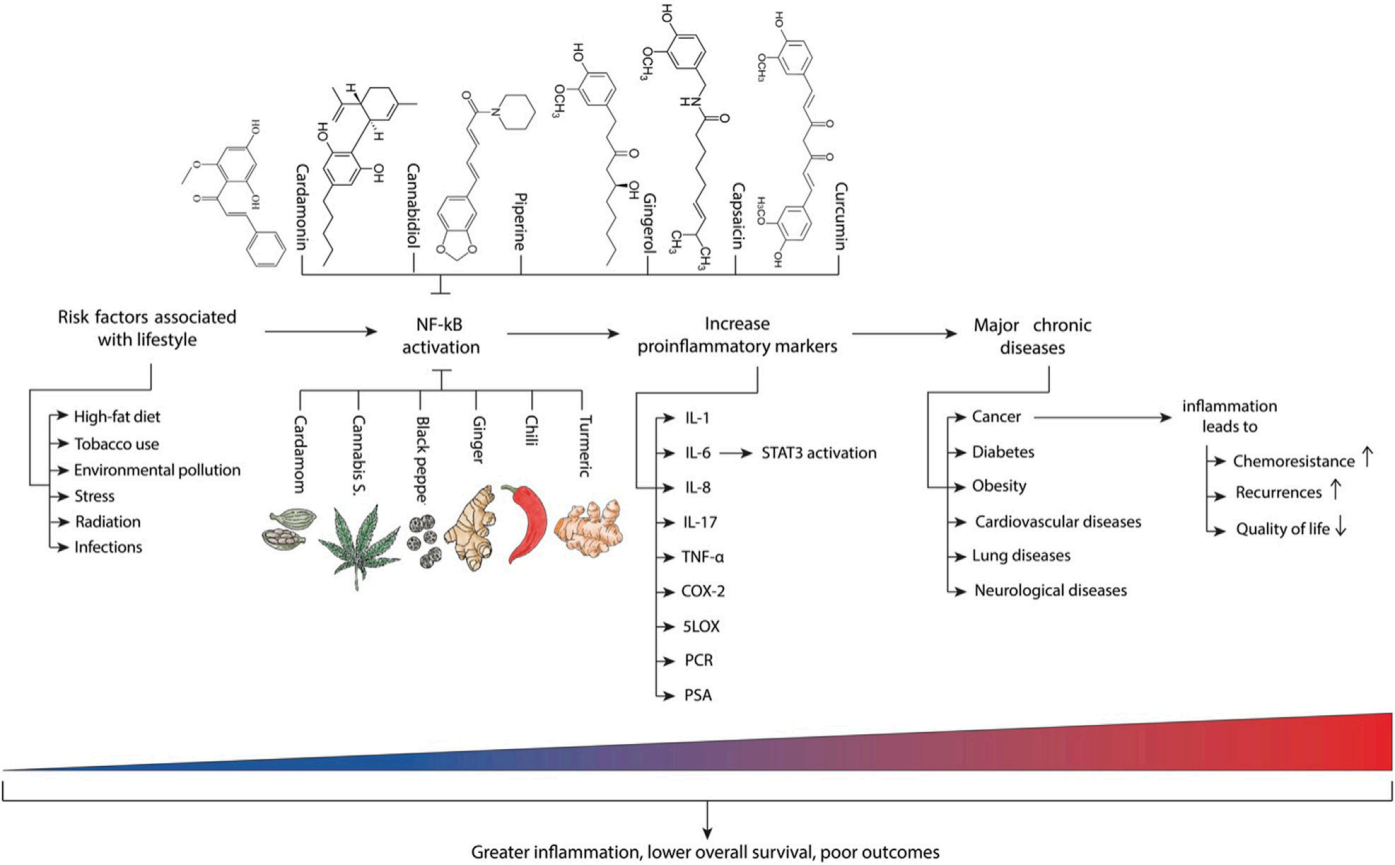
Main risk factors and inflammatory molecules associated with chronic diseases. Adapted from (Gupta et al., 2018b) with additional elements from (Suryavanshi et al., 2021).

An alternative therapeutic approach to modulate chronic inflammatory process involves the use of plant-derived products, which enclose a diverse array of compounds with pleiotropic properties capable of impacting molecular pathways common to both inflammatory phenomena and cancer (Garcia et al., 2014; Cragg and Pezzuto, 2016). Regular inclusion of these compounds in the diet, or their use in the treatment of established diseases has demonstrated potential in preventing inflammation (Figure 5).

Many cancer patients incorporate plant-derived products into their regimen during antineoplastic therapy, either to mitigate the side effects of conventional therapy or as adjuvants to enhance therapeutic outcomes (Desai et al., 2018). An illustrative example of the pleiotropic properties of plant-derived products is curcumin, a derivative of *Curcuma longa* which can modulate the arachidonic acid pathway (Yarla et al., 2016). Curcumin negatively regulates COX-2 expression by inhibiting NF-κB (Hong et al., 2004; Garcia et al., 2014). Furthermore, curcumin modulates the AKT/mTOR pathway and the expression of MMP-9 (Shishodia et al., 2003), a member of the MMP family regulating metastasis development (Chatterjee et al., 2018).

Despite promising results obtained with curcumin in animal models and humans, including encouraging effects on overall survival in patients with pancreatic and non-small cell lung cancer, further studies are needed to clearly define its utility in cancer treatment (Bahadori and Demiray, 2017). It is noteworthy that several MMP inhibitors like marimastat and tanomastat initially showing effectiveness comparable to conventional chemotherapeutics, faced challenges in clinical trials due to lack of selectivity and side effects (Nelson et al., 2017; Desai et al., 2018), such as musculoskeletal toxicity (Bramhall et al., 2001; Cathcart et al., 2015; Chatterjee et al., 2018). The pursuit of well-defined therapeutic profiles and safety considerations remains crucial for the development of effective plant-derived products in cancer treatment.

## 7 Endocannabinoid system (ECS)

The endocannabinoid system involves a complex interplay of components, which include lipid endocannabinoids (ECs) such as anandamide (AEA) and 2-arachidonoylglycerol (2-AG), synthesizing enzymes like NAPE-PLC and DAGL, hydrolyzing enzymes like FAAH and MAGL, and relevant receptors like a cannabinoid receptor-1 (CB1R), cannabinoid receptor-1 (CB2R), TRPV1, PPARγ, and GPR55 (Volkow et al., 2017). This system is ubiquitously distributed throughout the body, exerting diverse modulatory functions on various organs. Its notable impact on regulating food intake, perceiving pain, managing stress responses, influencing glucose tolerance, and controlling cell proliferation, differentiation, and metabolism highlights its potential therapeutic effect in cancer treatment (Pacher et al., 2020).

Alterations in the endocannabinoid system have been implicated in various diseases, including neurodegenerative disorders, multiple sclerosis, inflammation, and cancer (Laezza et al., 2020). Research has underscored the potential therapeutic effects of ECs in cancer treatment, with reports indicating antitumor effects in various cancer types, including breast cancer (Laezza et al., 2006), CRC (Lee et al., 2022; Silva-Reis et al., 2023), gliomas (Salazar et al., 2009; Costas-Insua and Guzmán, 2023), and others (Pisanti et al., 2013; Pagano et al., 2023).

Research indicates that conditions such as hypoxic stress can affect the endocannabinoid system, potentially increasing malignant activity in cells, such as glioblastoma cells. This effect is seen through the downregulation of CB1R and the upregulation of COX2 receptors (Sugimoto et al., 2017). Additionally, both physical and emotional stressors in these conditions can induce the release of adrenal corticosteroid hormones in both rodents and humans (Joëls, 2018). Elevated corticosteroids levels in serum have been linked to changes in the endocannabinoid system (Wamsteeker et al., 2010). Studies such as those conducted by Sugimoto et al. (2019), suggest that corticosterone may counteract the beneficial antitumor effects of ECs by reducing CB1R activity in glioblastoma models, thus promoting cancer growth. Given that stress is common in many diseases and corticosteroids are commonly used in treating inflammation and cancer, there is a debate about whether monitoring endocannabinoid levels could provide insights into disease progression by regulating their activity.

**FIGURE 6:**
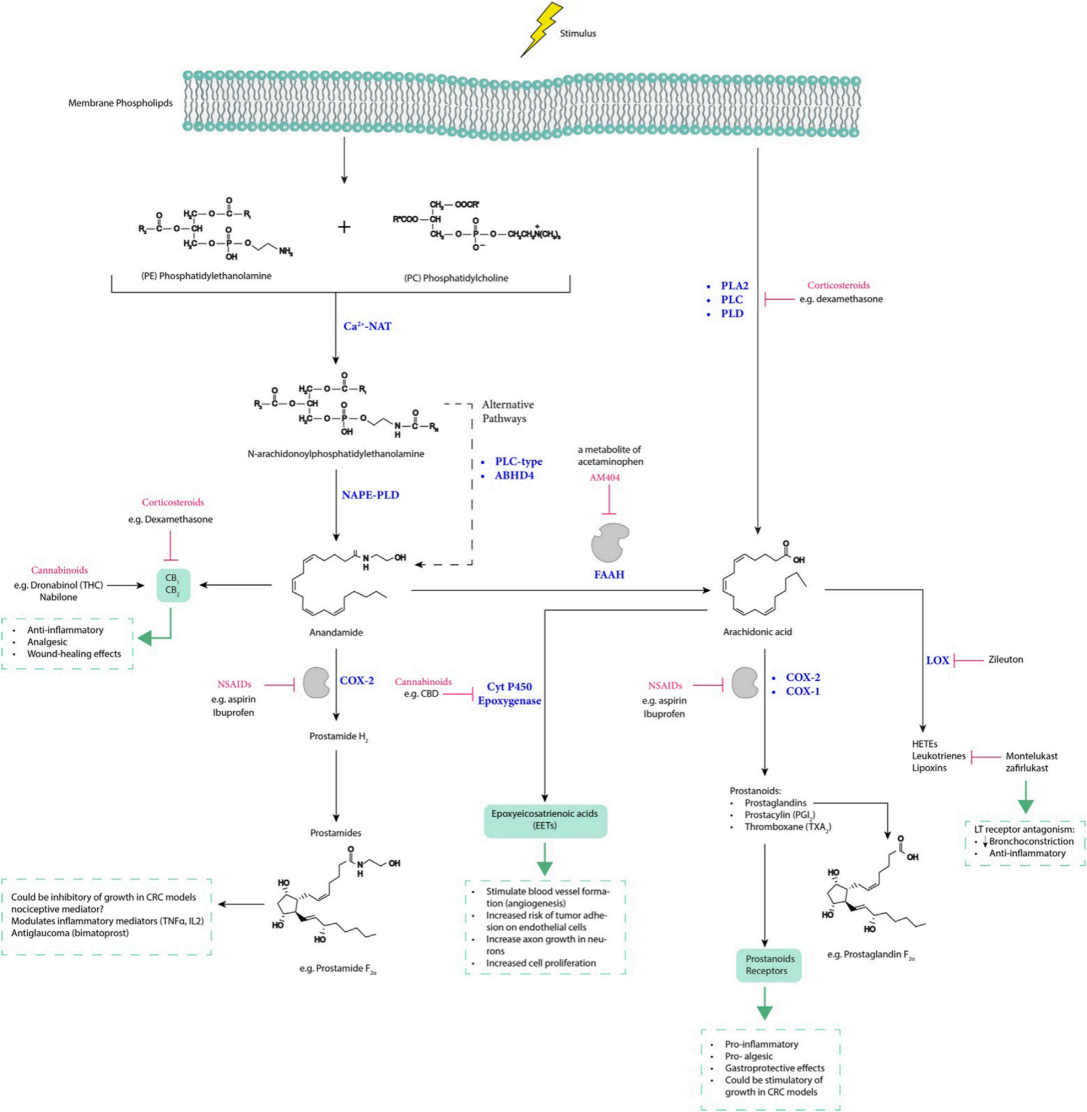
The interplay between arachidonic acid, endocannabinoids, and prostamides, along with drugs that modulate their expression, influences inflammatory-related diseases. LT receptor, leukotrienes receptor; LOX, lipoxygenase; CBD, cannabidiol; THC, tetrahydrocannabinol; NAPE-PLD, N-acylphosphatidylethanolamine-specific phospholipase D; PLC, phospholipase C; ABHD4, α/β-hydrolase domain-containing 4; AM404, N-arachidonoylphenolamine; Ca^2+−^NAT, calcium-dependent N-acyltransferase; PLA2, phospholipase A2; PLD, phospholipase D; FAAH, fatty acid amide hydrolase; and HETEs, hydroxyeicosatetraenoic acids.

The evidence suggests that cannabinoids are potent anti-inflammatory agents for treating chronic inflammatory diseases, although the precise mechanisms are not fully understood. Many chronic inflammatory conditions, including Coronavirus Disease 2019 (COVID-19), involve a phenomenon called the cytokine storm, where there is an uncontrolled and severe inflammatory response leading to the accumulation of proinflammatory cytokines (Suryavanshi et al., 2022). Modulating the endocannabinoid system using cannabis derivatives and other medications (Romano and Lograno, 2007; Park et al., 2016; Mallet et al., 2023), presents a promising alternative for treating and mitigating such situations. This approach, as illustrated in Figure 6, could offer additional therapeutic benefits.

## 8 Anti-inflammatory and anticancer effects of cannabinoids

Medical cannabinoids have demonstrated effectiveness in treating a range of diseases including Inflammatory Bowel Disease (IBD) (National Academies of Sciences, 2017), neurodegenerative disorders (Golub and Reddy, 2021), cancer (Mangal et al., 2021), anorexia, and weight loss (Bajtel et al., 2022; Pennant and Hinton, 2023). In the context of cancer, cannabinoids show promise as anticancer agents through several mechanisms:

1. Induction of apoptotic death in glioma (Velasco et al., 2016), hepatocellular carcinoma (Vara et al., 2011) and pancreatic cancer cells (Carracedo et al., 2006), by stimulating the synthesis of ceramide via CB1 and CB2 receptors. This process involves the upregulation of stress-regulated proteins, leading to inhibition of pathways promoting autophagy-mediated cell death.
2. Reduction of HIF-1α expression in Glioblastoma cells, resulting in decreased cancer cell growth and proliferation (Solinas et al., 2013).
3. Delayed tumor progression in human colon cancer cells by inhibiting HIF-1α expression and its target genes VEGF and COX-2 (Thapa et al., 2012), as well as activation of AMPK-related kinases to regulate energy metabolism in cancer cells (Dando et al., 2013).
4. Inhibition of cell proliferation and migration through GPR55 antagonism, leading to suppression of pathways promoting cancer cell survival and mobility (Pellati et al., 2018).

These findings suggest that cannabinoids hold potential as therapeutic agents against cancer, offering multiple avenues for intervention in cancer progression.

Cannabinoid medicines have gained approval from regulatory agencies including the United States Food and Drug Administration (FDA), as listed in Table 5. Notably, Phyto cannabinoids like Cannabidiol (CBD) and Tetrahydrocannabinol (Dronabinol, THC) have shown significant inhibition of NLRP3 inflammasome activation in macrophages following LPS + ATP stimulation. This leads to reduced levels of IL-1β. CBD also reduced NF-κB nuclear factor phosphorylation, while both CBD and THC mitigated post-LPS oxidative stress.

**TABLE 5:** Cannabis-based pharmaceutical drugs approved and marketed to 2023.

Active pharmaceutical ingredient	Drug name	Indication	Reference
Dronabinol (synthetic THC)	Marinol^®^/Syndros^®^	Antiemetic associated with chemotherapy and HIV/AIDS-induced anorexia	FDA (1985), Silvinato et al. (1992)
Cannabidiol	Epidiolex^®^	Dravet syndrome and Lennox-Gastaut Seizures associated with tuberous sclerosis complex	FDA (2017), Golub and Reddy (2021)
Nabilone (synthetic analog of THC)	Cesamet^®^	Chemotherapy-induced nausea and vomiting	CESAMET Nabilone Capsules (2022)
CBD:THC (Nabiximols 1:1 Plant-derived)	Sativex^®^	Multiple sclerosis (MS) related spasticity and Pain associated with MS and cancer	Keating (2017), Health Canada (2019)

ELISA data reveals substantial reductions in IL-6, IL-8, and tumor necrosis factor α (TNF-α) levels in macrophages following LPS treatment. Additionally, both CBD and THC significantly reduce STAT3 phosphorylation in macrophages, attributed to decreased phosphorylation of tyrosine kinase-2 (TYK2) after LPS stimulation (Desai et al., 2018).

Another phytocannabinoid, tetrahydrocannabivarin (THCV) downregulates IL-1β, IL-6, TNFα, and COX-2 at the protein level, possibly due to its effects on various stages of gene expression. At concentrations of 5 μM and 15 μM, THCV effectively inhibits NF-κB activation, which controls the transcription of numerous proinflammatory proteins including Pro-IL-1β, IL-6, Pro-TNFα, and COX-2 (Gojani et al., 2023).

Evidence suggests that CBD can suppress EGF/EGFR signaling, and downstream targets such as NF-κB, play roles in inflammation and cell growth regulation (Pellati et al., 2018). Additionally, it has been demonstrated that CBD and CBG can ameliorate inflammation in animal models of IBD (Borrelli et al., 2013; Hasenoehrl et al., 2016). Given the close association between IBD and an increased risk of CRC (Lucafò et al., 2021), recent research by Jeong et al. (2019), demonstrated the CBD’s ability to inhibit the induced dose-dependent growth and apoptosis of human CRC cells, while sparing normal colorectal cells. Additionally, CBD reduced tumor volume and stimulated apoptosis in a xenograft model, indicating its potential efficacy as a reliable anticancer agent *in vivo* (Jeong et al., 2019). Furthermore, a pilot study by Ng et al. (2023) involving three CRC patients, revealed that THC could alleviate CRC cachexia associated with inflammation and immune responses. After THC treatment, a decrease was observed in the level of six cytokines in serum samples, including IL-6 and TNF-α, as well as chemokine (C-C motif) ligand 2 (CCL2), known for its unfavorable effects on tumor prognosis (Jin et al., 2021), and reviewed elsewhere (Xu et al., 2021). These findings collectively underscore the promising therapeutic potential of cannabinoids in addressing inflammation and combating CRC progression.

Cannabichromene (CBC) interacts with CB1 and CB2 receptors, influences cellular endocannabinoid reuptake and acts as an agonist on TRPA1 and adenosine receptors. These actions lead to downstream effects such as suppression of MAGL function, decreased intracellular nitric oxide (NO) and IFNγ levels in macrophages, and modulation of cytokine and COX-2 gene transcription. The precise mechanisms underlying CBC’s anti-inflammatory properties involve both CB1R and CB2R mediated signaling as well as TRPA1 agonism (Udoh et al., 2019), which helps mitigate inflammatory responses associated with cytokine and COX-2 transcriptional modulation (Gojani et al., 2023). On the other hand, Cannabinol (CBN) modulates NFκB activation primarily by regulating phosphorylation rather than transcription. It has a dose-dependent effect on P-NFκB levels, with lower doses yielding more pronounced effects. While CBN upregulated the transcription of COX-2 and Pro-TNFα genes, it simultaneously decreases their protein levels, suggesting a potential post-transcriptional mode of action. Additionally, CBN exhibited inhibitory effects on IL-1β and IL-6 gene transcription, likely mediated by negative regulation of NF-κB phosphorylation and activation (Gojani et al., 2023).

Overall, both CBC and CBN demonstrated diverse anti-inflammatory properties through distinct mechanisms. CBCs effect appear to involve modulation of cannabinoid reuptake TRPA-1 agonism and CB receptor signaling, while CBN primarily exerts its effects through regulation of NFκB phosphorylation and potential post-transcriptional mechanisms (Gojani et al., 2023). These findings support the potential use of cannabinoids as anti-inflammatory agents for various chronic inflammatory diseases and provide insights into their mechanisms of action.

## 9 Analysis and conclusion

This extensive review highlights the urgent need for further research to discover valuable biomarkers for cancer diagnosis, prevention, and treatment. The relationship between obesity, chronic inflammation, and cancer emphasizes the importance of population-based interventions, including the maintenance of healthy body weight as a preventive measure (Kolb et al., 2016; Lauby-Secretan et al., 2016). Moreover, the association between the dietary inflammatory index and increased cancer risk suggest the potential effectiveness of an anti-inflammatory diet supplemented with nutraceuticals (Jayedi et al., 2018) and coupled with a healthy lifestyle for cancer prevention (Michels et al., 2021).

Furthermore, the characteristics of senescence and the impact of SASP on TME provide opportunities for developing improved therapeutic strategies. Understanding the vulnerabilities associated with senescence and the inflammatory phenomena linked to carcinogenesis can inform targeted interventions. The use of natural products and their derivatives emerges as a promising therapeutic alternative or as an adjuvant for cancer-related inflammation, often presenting a more favorable adverse effect profile than conventional therapeutic agents (Deng et al., 2017; Desai et al., 2018; Deng et al., 2020). However, the development of drugs based on bioactive natural products poses challenges due to their inherent variability, complexity and the need for standardization and rigorous research processes (Desai et al., 2018; Huang et al., 2021).

In addition, anti-inflammatory products, including NSAIDs (Zappavigna et al., 2020), corticosteroids (Wang et al., 2004), and statins, (Masjedi et al., 2021), along with plant-derived products hold clinical utility as adjuvants to traditional therapies. Their potential accessibility and known safety profiles, make them an attractive alternative for the general population. Combining anti-inflammatory drugs with adjustments in dosage and dosing regimens could represent a novel strategy in antitumor therapy (Binnewies et al., 2018; Zappavigna et al., 2020).

Moreover, the exploration of endocannabinoid, cannabinoid, and prostamide-type compounds as potential therapeutic targets or biomarkers of inflammatory and immune processes in tumor development requires further investigation to elucidate their pharmacology and significance before transitioning to clinical practice. Recent evidence suggests that cannabinoids may inhibit tumor cell proliferation and serve as supportive therapy for anti-tumor treatments due to their multiple therapeutic targets contributing to anti-neoplastic effects. Ongoing clinical studies with nabiximols (Sativex) indicate promising potential in the treatment of glioblastoma. The first, known as ARISTOCRAT (NCT05629702), is a phase II, multi-center, double-blind, placebo-controlled, randomized trial aimed at comparing the cannabinoid Nabiximols with a placebo in patients with recurrent MGMT methylated glioblastoma (GBM) treated with temozolomide (TMZ). The second study (NCT03529448) is a Phase Ib, open-label, multicenter, intrapatient dose-escalation clinical trial designed to assess the safety profile of the (THC:CBD 1:1) combination with temozolomide and radiotherapy in patients newly diagnosed with glioblastoma.

Understanding factors like genetic variability, environmental exposure history, dietary habits, microbiome composition, and cellular plasticity within the TME, promises insights into the intricate interrelationships governing carcinogenic phenomena and may contribute to the discovery of diagnostic or clinically relevant biomarkers (Greten and Grivennikov, 2019; Martin et al., 2021), establishing possible therapies based on molecular and genetic analysis in patients.

In conclusion, a Poly pharmacological approach to modulate the complex signaling of inflammation and cancer appears to be the most promising option. Given the systemic and multifactorial nature of inflammation (Hou et al., 2021), targeting multiple proteins involved in inflammation and cancer pathogenesis is deemed more effective than focusing on a single gene, protein, or signaling pathway. While short-term treatments may benefit from targeting a single therapeutic target, a comprehensive strategy that addresses the persistent nature of inflammation and the involvement of multiple signaling pathways in cancer cells is crucial for long-term success.

## Abbreviations

NSAIDs, non-steroidal anti-inflammatory drugs; BAX, Bcl-2-associated protein X; CRC, colorectal cancer; COX, cyclooxygenase enzymes; ECs, endocannabinoids; gp130, glycoprotein 130; IL-6, interleukin-6; IL-10, interleukin-10; JAK, janus kinase; NF-κB, nuclear factor-κB; MMP, matrix metalloproteinases; STAT, signal transducer and activator of transcription; STAT3, signal transducer and activator of transcription 3; TAM, tumor-associated macrophages; TME, tumor microenvironment; TNFα, tumor necrosis factor-α; HPV, human papillomavirus.

## References

[B1] Aarnoutse R. Ziemons J. Penders J. Rensen S. S. de Vos-Geelen J. Smidt M. L. (2019). The clinical link between human intestinal microbiota and systemic cancer therapy. Int. J. Mol. Sci. 20 (17), 4145. 10.3390/ijms20174145 31450659 PMC6747354

[B2] Algra A. M. Rothwell P. M. (2012). Effects of regular aspirin on long-term cancer incidence and metastasis: a systematic comparison of evidence from observational studies versus randomised trials. Lancet Oncol 13 (5), 518–527. 10.1016/S1470-2045(12)70112-2 22440112

[B3] Ammirante M. Shalapour S. Kang Y. Jamieson C. A. M. Karin M. (2014). Tissue injury and hypoxia promote malignant progression of prostate cancer by inducing CXCL13 expression in tumor myofibroblasts. Proc. Natl. Acad. Sci. U. S. A. 111 (41), 14776–14781. 10.1073/pnas.1416498111 25267627 PMC4205637

[B4] Ancrile B. Lim K. Counter C. (2007). Oncogenic Ras-induced secretion of IL6 is required for tumorigenesis. Genes. Dev. 21 (14), 1714–1719. 10.1101/gad.1549407 17639077 PMC1920165

[B5] Anglesio M. George J. Kulbe H. Friedlander M. Rischin D. Lemech C. (2011). IL6-STAT3-HIF signaling and therapeutic response to the angiogenesis inhibitor sunitinib in ovarian clear cell cancer. Clin. Cancer Res. 17 (8), 2538–2548. 10.1158/1078-0432.CCR-10-3314 21343371

[B6] Bahadori F. Demiray M. (2017). A realistic view on “the essential medicinal chemistry of curcumin”. ACS Rev. Med. Chem. Lett. 8 (9), 893–896. 10.1021/acsmedchemlett.7b00284 PMC5601379 28947929

[B7] Bajtel Á. Kiss T. Tóth B. Kiss S. Hegyi P. Vörhendi N. (2022). The safety of dronabinol and nabilone: a systematic review and meta-analysis of clinical trials. Pharm. (Basel) 15 (1), 100. 10.3390/ph15010100 PMC8778752 35056154

[B8] Balistreri C. R. Caruso C. Candore G. (2010). The role of adipose tissue and adipokines in obesity-related inflammatory diseases. Mediat. Inflamm. 2010, 802078. 10.1155/2010/802078 PMC2910551 20671929

[B9] Barabutis N. Schally A. v. Siejka A. (2018). P53, GHRH, inflammation and cancer. EBioMedicine 37, 557–562. 10.1016/j.ebiom.2018.10.034 30344124 PMC6284454

[B10] Becatti M. Mannucci A. Taddei N. Fiorillo C. (2018). Oxidative stress and inflammation: new molecular targets for cardiovascular diseases. Intern Emerg. Med. 13 (5), 647–649. 10.1007/s11739-018-1865-3 29858969

[B11] Ben-Neriah Y. Karin M. (2011). Inflammation meets cancer, with NF-κB as the matchmaker. Nat. Immunol. 12 (8), 715–723. 10.1038/ni.2060 21772280

[B12] Bhavsar N. A. Bream J. H. Meeker A. K. Drake C. G. Peskoe S. B. Dabitao D. (2014). A peripheral circulating TH1 cytokine profile is inversely associated with prostate cancer risk in CLUE II. Cancer Ep Prev Bio Prev. 23 (11), 2561–2567. 10.1158/1055-9965.EPI-14-0010 PMC4648294 25150281

[B13] Binnewies M. Roberts E. W. Kersten K. Chan V. Fearon D. F. Merad M. (2018). Understanding the tumor immune microenvironment (TIME) for effective therapy. Nat. Med. 24 (5), 541–550. 10.1038/s41591-018-0014-x 29686425 PMC5998822

[B14] Borrelli F. Fasolino I. Romano B. Capasso R. Maiello F. Coppola D. (2013). Beneficial effect of the non-psychotropic plant cannabinoid cannabigerol on experimental inflammatory bowel disease. Biochem. Pharmacol. 85 (9), 1306–1316. 10.1016/j.bcp.2013.01.017 23415610

[B15] Bramhall S. R. Rosemurgy A. Brown P. D. Bowry C. Buckles J. A. C. Marimastat Pancreatic Cancer Study Group (2001). Marimastat as first-line therapy for patients with unresectable pancreatic cancer: a randomized trial. J Clin. Oncol. 19 (15), 3447–3455. 10.1200/JCO.2001.19.15.3447 11481349

[B16] Brown K. L. Davidson J. Rotondo D. (2013). Characterisation of the prostaglandin E2-ethanolamide suppression of tumour necrosis factor-α production in human monocytic cells. Biochim. Biophys. Acta 1831 (6), 1098–1107. 10.1016/j.bbalip.2013.03.006 23542062

[B17] Brüser A. Zimmermann A. Crews B. C. Sliwoski G. Meiler J. König G. M. (2017). Prostaglandin E2 glyceryl ester is an endogenous agonist of the nucleotide receptor P2Y6. Sci. Rep. 7 (1), 2380. 10.1038/s41598-017-02414-802414-8 28539604 PMC5443783

[B18] Caliri A. W. Tommasi S. Besaratinia A. (2021). Relationships among smoking, oxidative stress, inflammation, macromolecular damage, and cancer. Mut. Res. Rev. Mutat. Res. 787, 108365. 10.1016/j.mrrev.2021.108365 34083039 PMC8287787

[B19] Candido J. Hagemann T. (2013). Cancer-related inflammation. J. Clin. Immunol. 33 (Suppl. 1), S79–S84. 10.1007/s10875-012-9847-0 23225204

[B20] Capece D. Verzella D. Tessitore A. Alesse E. Capalbo C. Zazzeroni F. (2018). Cancer secretome and inflammation: the bright and the dark sides of NF-κB. Semin. Cell. Dev. Biol. 78, 51–61. 10.1016/j.semcdb.2017.08.004 28779979

[B21] Carracedo A. Gironella M. Lorente M. Garcia S. Guzmán M. Velasco G. (2006). Cannabinoids induce apoptosis of pancreatic tumor cells via endoplasmic reticulum stress-related genes. Cancer Res. 66 (13), 6748–6755. 10.1158/0008-5472.CAN-06-0169 16818650

[B22] Cathcart J. Pulkoski-Gross A. Cao J. (2015). Targeting matrix metalloproteinases in cancer: bringing new life to old ideas. Genes. & Dis. 2 (1), 26–34. 10.1016/j.gendis.2014.12.002 PMC4474140 26097889

[B23] CESAMET (Nabilone) Capsules (2022) Nda 18-677/S-011. Available at: https://www.accessdata.fda.gov/drugsatfda_docs/label/2006/018677s011lbl.pdf.

[B24] Chatterjee K. Jana S. Choudhary P. Swarnakar S. (2018). Triumph and tumult of matrix metalloproteinases and their crosstalk with eicosanoids in cancer. Cancer Metastasis Rev. 37 (2), 279–288. 10.1007/s10555-018-9756-7 30094569

[B25] Chen Z. Hsieh Y. Huang C. Tsai C. (2017). Inhibitory effects of probiotic lactobacillus on the growth of human colonic carcinoma cell line HT-29. Molecules 22 (1), 107. 10.3390/molecules22010107 28075415 PMC6155858

[B26] Cheng Y. Ling Z. Li L. (2020). The intestinal microbiota and colorectal cancer. Front. Immunol. 11, 615056. 10.3389/fimmu.2020.615056 33329610 PMC7734048

[B27] Cildir G. Akincilar S. C. Tergaonkar V. (2013). Chronic adipose tissue inflammation: all immune cells on the stage. Trends Mol. Med. 19 (8), 487–500. 10.1016/j.molmed.2013.05.001 23746697

[B28] Cnop M. Foufelle F. Velloso L. A. (2012). Endoplasmic reticulum stress, obesity and diabetes. Trends Mol. Med. 18 (1), 59–68. 10.1016/j.molmed.2011.07.010 21889406

[B29] Colotta F. Allavena P. Sica A. Garlanda C. Mantovani A. (2009). Cancer-related inflammation, the seventh hallmark of cancer: links to genetic instability. Carcinogenesis 30 (7), 1073–1081. 10.1093/carcin/bgp127 19468060

[B30] Costas-Insua C. Guzmán M. (2023). Endocannabinoid signaling in glioma. Glia 71 (1), 127–138. 10.1002/glia.24173 35322459 PMC9790654

[B31] Cragg G. M. Pezzuto J. M. (2016). Natural products as a vital source for the discovery of cancer chemotherapeutic and chemopreventive agents. Med Princ. Pract. 25 (Suppl. 2), 41–59. 10.1159/000443404 26679767 PMC5588531

[B32] Crusz S. M. Balkwill F. R. (2015). Inflammation and cancer: advances and new agents. Nat. Rev. Clin. Oncol. 12 (10), 584–596. 10.1038/nrclinonc.2015.105 26122183

[B33] Dajee M. Lazarov M. Zhang J. Y. Cai T. Green C. L. Russell A. J. (2003). NF-kappaB blockade and oncogenic Ras trigger invasive human epidermal neoplasia. Nature 421 (6923), 639–643. 10.1038/nature01283 12571598

[B34] Dando I. Donadelli M. Costanzo C. Dalla Pozza E. D'Alessandro A. Zolla L. (2013). Cannabinoids inhibit energetic metabolism and induce AMPK-dependent autophagy in pancreatic cancer cells. Cell. Death Dis. 4 (6), e664. 10.1038/cddis.2013.151 23764845 PMC3698539

[B35] Del Carmen S. De Moreno De LeBlanc A. Levit R. Azevedo V. Langella P. Bermúdez-Humarán L. (2017). Anti-cancer effect of lactic acid bacteria expressing antioxidant enzymes or IL-10 in a colorectal cancer mouse model. Int. Immunopharmacol. 42, 122–129. 10.1016/j.intimp.2016.11.017 27912148

[B36] Deng L. J. Qi M. Li N. Lei Y. H. Zhang D. M. Chen J. X. (2020). Natural products and their derivatives: promising modulators of tumor immunotherapy. J Leukoc. Biol. 108 (2), 493–508. 10.1002/JLB.3MR0320-444R 32678943 PMC7496826

[B37] Deng L. J. Wang L. H. Peng C. K. Li Y. bin Huang M. H. Chen M. F. (2017). Fibroblast activation protein α activated tripeptide bufadienolide antitumor prodrug with reduced cardiotoxicity. J. Med. Chem. 60 (13), 5320–5333. 10.1021/acs.jmedchem.6b01755 28595013

[B38] Desai S. Prickril B. Rasooly A. (2018). Mechanisms of phytonutrient modulation of Cyclooxygenase-2 (COX- 2) and inflammation related to cancer. Nutr. Cancer 70 (3), 350–375. 10.1080/01635581.2018.1446091 29578814 PMC6309701

[B39] DiDonato J. Mercurio F. Karin M. (2012). NF-κB and the link between inflammation and cancer. Immunol. Rev. 246 (1), 379–400. 10.1111/j.1600-065X.2012.01099.x 22435567

[B40] Escobar G. Orozco A. Núñez J. Muñoz F. Escobar G. Orozco A. (2020). Mortalidad por enfermedades cardiovasculares en Colombia 1993-2017. Un análisis de las políticas públicas. Rev. Salud Uni 36 (3), 558–570. 10.14482/sun.36.3.616.12

[B41] Eugen-Olsen J. Andersen O. Linneberg A. Ladelund S. Hansen T. W. Langkilde A. (2010). Circulating soluble urokinase plasminogen activator receptor predicts cancer, cardiovascular disease, diabetes and mortality in the general population. J Intern. Med. 268 (3), 296–308. 10.1111/j.1365-2796.2010.02252.x 20561148

[B42] Fasshauer M. Blüher M. (2015). Adipokines in health and disease. Trends Pharmacol. Sci. 36 (7), 461–470. 10.1016/j.tips.2015.04.014 26022934

[B43] FDA (1985) Marinol label, NDA 18-651. Available at: https://www.accessdata.fda.gov/scripts/cder/daf/index.cfm?event=overview.process&ApplNo=018651 (Accessed March 13, 2024).

[B44] FDA (2017). Drug label information: SYNDROS. Available at: https://www.accessdata.fda.gov/drugsatfda_docs/label/2017/205525s003lbl.pdfhttps://www.accessdata.fda.gov/drugsatfda_docs/label/2017/205525s003lbl.pdf (Accessed March 13, 2024).

[B45] Ferlay J. Colombet M. Soerjomataram I. Parkin D. M. Piñeros M. Znaor A. (2021). Estimating the global cancer incidence and mortality in 2018: GLOBOCAN sources and methods. Int. J. Cancer 144, 1941–1953. 10.1002/ijc.31937 30350310

[B46] Galdiero M. R. Marone G. Mantovani A. (2018). Cancer inflammation and cytokines. Cold Spring Harb. Perspect. Biol. 10 (8), a028662. 10.1101/cshperspect.a028662 28778871 PMC6071493

[B47] Garcia R. Yan M. Search D. Zhang R. Carson N. Ryan C. (2014). P2Y6 receptor potentiates pro-inflammatory responses in macrophages and exhibits differential roles in atherosclerotic lesion development. PloS One 9 (10), e111385. 10.1371/journal.pone.0111385 25360548 PMC4216081

[B48] Gargalionis A. Papavassiliou K. Papavassiliou A. (2021). Targeting STAT3 signaling pathway in colorectal cancer. Biomedicines 9 (8), 1016. 10.3390/biomedicines9081016 34440220 PMC8392110

[B49] Ghiringhelli F. Fumet J. D. (2019). Is there a place for immunotherapy for metastatic microsatellite stable colorectal cancer? Front Immunol 10, 1816. 10.3389/fimmu.2019.01816 31447840 PMC6691024

[B50] Giannattasio G. Ohta S. Boyce J. Xing W. Balestrieri B. Boyce J. (2011). The purinergic G protein-coupled receptor 6 inhibits effector T cell activation in allergic pulmonary inflammation. J. Immunol. 187 (3), 1486–1495. 10.4049/jimmunol.1003669 21724990 PMC3140636

[B51] Glass M. Hong J. Sato T. Mitchell M. (2005). Misidentification of prostamides as prostaglandins. J. Lipid Res. 46 (7), 1364–1368. 10.1194/jlr.C500006-JLR200 15863842

[B52] Gojani E. Wang B. Li D. Kovalchuk O. Kovalchuk I. (2023). Anti-inflammatory effects of minor cannabinoids CBC, THCV, and CBN in human macrophages. Molecules 28 (18), 6487. 10.3390/molecules28186487 37764262 PMC10534668

[B53] Golub V. Reddy D. S. (2021). Cannabidiol therapy for refractory epilepsy and seizure disorders. Adv. Exp. Med. Biol. 1264, 93–110. 10.1007/978-3-030-57369-0_7 33332006

[B54] Gouveia-Figueira S. Nording M. L. (2015). Validation of a tandem mass spectrometry method using combined extraction of 37 oxylipins and 14 endocannabinoid-related compounds including prostamides from biological matrices. Prostagl. Other Lipid Mediat 121 (Pt A), 110–121. 10.1016/j.prostaglandins.2015.06.003 26115647

[B55] Gregor M. F. Hotamisligil G. S. (2011). Inflammatory mechanisms in obesity. Annu. Rev. Immunol. 29, 415–445. 10.1146/annurev-immunol-031210-101322 21219177

[B56] Greten F. R. Grivennikov S. I. (2019). Inflammation and cancer: triggers, mechanisms and consequences. Immunity 51 (1), 27–41. 10.1016/j.immuni.2019.06.025 31315034 PMC6831096

[B57] Grivennikov S. I. Greten F. R. Karin M. (2010). Immunity, inflammation, and cancer. Cell. 140 (6), 883–899. 10.1016/j.cell.2010.01.025 20303878 PMC2866629

[B58] Guha A. Wang X. Harris R. Nelson A. Stepp D. Klaassen Z. (2021). Obesity and the bidirectional risk of cancer and cardiovascular diseases in African Americans: disparity vs. Ancestryancestry. Front. Card Med 08, 1360761488. 10.3389/fcvm.2021.761488 PMC8558482 34733899

[B59] Gupta S. C. Kunnumakkara A. B. Aggarwal S. Aggarwal B. B. (2018a). Inflammation, a double-edge sword for cancer and other age-related diseases. Front. Immunol. 9, 2160. 10.3389/fimmu.2018.02160 30319623 PMC6170639

[B60] Gupta S. C. Kunnumakkara A. B. Aggarwal S. Aggarwal B. B. (2018b). Inflammation, a double-edge sword for cancer and other age-related diseases. J. Med. 9, 2160. 10.3389/fimmu.2018.02160 PMC6170639 30319623

[B61] Han J. M. Levings M. K. (2013). Immune regulation in obesity-associated adipose inflammation. J. Immunol. 191 (2), 527–532. 10.4049/jimmunol.1301035 23825387

[B62] Hanahan D. Coussens L. M. (2012). Accessories to the crime: functions of cells recruited to the tumor microenvironment. Cancer Cell. 21 (3), 309–322. 10.1016/j.ccr.2012.02.022 22439926

[B63] Hanahan D. Weinberg R. A. (2011). Hallmarks of cancer: the next generation. Cell. 144 (5), 646–674. 10.1016/j.cell.2011.02.013 21376230

[B64] Harbeck N. Gnant M. (2017). Breast cancer. Lancet 389 (10074), 1134–1150. 10.1016/S0140-6736(16)31891-8 27865536

[B65] Hasenoehrl C. Taschler U. Storr M. Schicho R. (2016). The gastrointestinal tract - a central organ of cannabinoid signaling in health and disease. Neurogastroenterol. Motil. 28 (12), 1765–1780. 10.1111/nmo.12931 27561826 PMC5130148

[B66] Health Canada (2019). “Product monograph: sativex oromucosal spray,” in Health Canada drug product database. Available at: https://pdf.hres.ca/dpd_pm/00016162.PDF.

[B67] Hill C. Guarner F. Reid G. Gibson G. R. Merenstein D. J. Pot B. (2014). Expert consensus document. The International Scientific Association for Probiotics and Prebiotics consensus statement on the scope and appropriate use of the term probiotic. Nat. Rev. Gastroenterol. Hepatol. 11, 506–514. 10.1038/nrgastro.2014.66 24912386

[B68] Hirano T. (2021a). IL-6 in inflammation, autoimmunity and cancer. Int. Immunol. 33 (3), 127–148. 10.1093/intimm/dxaa078 33337480 PMC7799025

[B69] Hirano T. (2021b). IL-6 in inflammation, autoimmunity and cancer. Int. Immunol. 33 (3), 127–148. 10.1093/intimm/dxaa078 33337480 PMC7799025

[B70] Hong J. Bose M. Ju J. Ryu J. H. Chen X. Sang S. (2004). Modulation of arachidonic acid metabolism by curcumin and related beta-diketone derivatives: effects on cytosolic phospholipase A (2), cyclooxygenases and 5-lipoxygenase. Carcinogenesis 25 (9), 1671–1679. 10.1093/carcin/bgh165 15073046

[B71] Hou J. Karin M. Sun B. (2021). Targeting cancer-promoting inflammation — have anti-inflammatory therapies come of age? Nat. Rev. Clin. Oncol. Oncol. 18 (5), 261–279. 10.1038/s41571-020-00459-9 PMC8978805 33469195

[B72] Hsu T. (2016). Educational initiatives in geriatric oncology - who, why, and how? J. Geriatr. Oncol. 7 (5), 390–396. 10.1016/j.jgo.2016.07.013 27567256

[B73] Huang M. Lu J. J. Ding J. (2021). Natural products in cancer therapy: past, present and future. Nat Prod. Bioprospecting. 11 (1), 5–13. 10.1007/s13659-020-00293-7 PMC7933288 33389713

[B74] Iida N. Dzutsev A. Stewart C. Smith L. Bouladoux N. Weingarten R. A. (2013). Commensal bacteria control cancer response to therapy by modulating the tumor microenvironment. Science. 342 (6161), 967–970. 10.1126/science.1240527 24264989 PMC6709532

[B75] Jayedi A. Emadi A. Shab-Bidar S. (2018). Dietary Inflammatory Index and site-specific cancer risk: a systematic review and dose-response meta-analysis. Adv. Nut 9 (4), 388–403. 10.1093/advances/nmy015 PMC6054175 30032224

[B76] Jeong S. Yun H. K. Jeong Y. A. Jo M. J. Kang S. H. Kim J. L. (2019). Cannabidiol-induced apoptosis is mediated by activation of Noxa in human colorectal cancer cells. Cancer Lett. 447, 12–23. 10.1016/j.canlet.2019.01.011 30660647

[B77] Jin J. Lin J. Xu A. Lou J. Qian C. Li X. (2021). CCL2: an important mediator between tumor cells and host cells in tumor microenvironment. Front. Oncol. 11, 722916. 10.3389/fonc.2021.722916 34386431 PMC8354025

[B78] Jin W. Xu J. Xu W. Gu D. Li P. (2014). Diagnostic value of interleukin-8 in colorectal cancer: a case-control study and meta-analysis. World J. Gastroenterol. 20 (43), 16334–16342. 10.3748/wjg.v20.i43.16334 25473192 PMC4239526

[B79] Joëls M. (2018). Corticosteroids and the brain. J. Endocrinol. 238, R121–R130. 10.1530/JOE-18-0226 29875162

[B80] Jorgensen I. Rayamajhi M. Miao E. A. (2017). Programmed cell death as a defence against infection. Nat. Rev. Immunol. 17 (3), 151–164. 10.1038/nri.2016.147 28138137 PMC5328506

[B81] Kantola T. Klintrup K. Väyrynen J. Vornanen J. Bloigu R. Karhu T. (2012). Stage-dependent alterations of the serum cytokine pattern in colorectal carcinoma. Br. J. Cancer 107 (10), 1729–1736. 10.1038/bjc.2012.456 23059742 PMC3493870

[B82] Keating G. M. (2017). Delta-9-Tetrahydrocannabinol/Cannabidiol oromucosal spray (Sativex®): a review in multiple sclerosis-related spasticity. Drugs 77 (5), 563–574. 10.1007/s40265-017-0720-6 28293911

[B83] Khandia R. Munjal A. (2020). Interplay between inflammation and cancer. Adv. Protein Chem. Struct. Biol. 119, 199–245. 10.1016/bs.apcsb.2019.09.004 31997769

[B84] Kim E. H. Wong S. W. Martinez J. (2019). Programmed necrosis and disease: we interrupt your regular programming to bring you necroinflammation. Cell. Death Dif 26 (1), 25–40. 10.1038/s41418-018-0179-3 PMC6294794 30349078

[B85] Kolb R. Sutterwala F. S. Zhang W. (2016). Obesity and cancer: inflammation bridges the two. Curr. Opin. Phl 29, 77–89. 10.1016/j.coph.2016.07.005 PMC4992602 27429211

[B86] Kozak K. Crews B. Morrow J. Wang L. Ma Y. Weinander R. (2002). Metabolism of the endocannabinoids, 2-arachidonylglycerol and anandamide, into prostaglandin, thromboxane, and prostacyclin glycerol esters and ethanolamides. J. Biol. Chem. 277 (47), 44877–44885. 10.1074/jbc.M206788200 12244105

[B87] Kratz M. Coats B. R. Hisert K. B. Hagman D. Mutskov V. Peris E. (2014). Metabolic dysfunction drives a mechanistically distinct proinflammatory phenotype in adipose tissue macrophages. Cell. Metab. 20 (4), 614–625. 10.1016/j.cmet.2014.08.010 25242226 PMC4192131

[B88] Kulbe H. Thompson R. Wilson J. Robinson S. Hagemann T. Fatah R. (2007). The inflammatory cytokine tumor necrosis factor-alpha generates an autocrine tumor-promoting network in epithelial ovarian cancer cells. Cancer Res. 67 (2), 585–592. 10.1158/0008-5472.CAN-06-2941 17234767 PMC2679985

[B89] Kumari N. Dwarakanath B. Das A. Bhatt A. (2016). Role of interleukin-6 in cancer progression and therapeutic resistance. Tumor Biol. 37 (9), 11553–11572. 10.1007/s13277-016-5098-75098-7 27260630

[B90] Laezza C. Pagano C. Navarra G. Pastorino O. Proto M. C. Fiore D. (2020). The endocannabinoid system: a target for cancer treatment. Int. J. Mol. Sci. 21, 747. 10.3390/ijms21030747 31979368 PMC7037210

[B91] Laezza C. Pisanti S. Crescenzi E. Bifulco M. (2006). Anandamide inhibits Cdk2 and activates Chk1 leading to cell cycle arrest in human breast cancer cells. FEBS Lett. 580 (26), 6076–6082. 10.1016/j.febslet.2006.09.074 17055492

[B92] Lauby-Secretan B. Scoccianti C. Loomis D. Grosse Y. Bianchini F. Straif K. (2016). Body fatness and cancer — viewpoint of the IARC working group. N. Engl. J Med. 375 (8), 794–798. 10.1056/NEJMsr1606602 27557308 PMC6754861

[B93] Lee H.-S. Tamia G. Song H.-J. Amarakoon D. Wei C.-I. Lee S.-H. (2022). Cannabidiol exerts anti-proliferative activity via a cannabinoid receptor 2-dependent mechanism in human colorectal cancer cells. Int. Immunopharmacol. 108, 108865. 10.1016/j.intimp.2022.108865 35598400

[B94] Lippitz B. E. Harris R. A. (2016). Cytokine patterns in cancer patients: a review of the correlation between interleukin 6 and prognosis. Oncoimmunology 5 (5), e1093722. 10.1080/2162402X.2015.1093722 27467926 PMC4910721

[B95] Liu P. H. Wu K. Ng K. Zauber A. G. Nguyen L. H. Song M. (2019). Association of obesity with risk of early-onset colorectal cancer among women. JAMA Oncol. 5 (1), 37–44. 10.1001/jamaoncol.2018.4280 30326010 PMC6382547

[B96] Loh C. Arya A. Naema A. Wong W. Sethi G. Looi C. (2019). Signal transducer and activator of transcription (STATs) proteins in cancer and inflammation: functions and therapeutic implication. Front. Oncol. 9, 48. 10.3389/fonc.2019.00048 30847297 PMC6393348

[B97] López-Otın C. Blasco M. A. Partridge L. Serrano M. Kroemer G. (2013). The hallmarks of aging. Cell. 153 (6), 1194–1217. 10.1016/j.cell.2013.05.0392013.05.039 23746838 PMC3836174

[B98] Lorincz A. M. Sukumar S. (2006). Molecular links between obesity and breast cancer. Endocr. Relat. Cancer 13 (2), 279–292. 10.1677/erc.1.00729 16728564

[B99] Lucafò M. Curci D. Franzin M. Decorti G. Stocco G. (2021). Inflammatory bowel disease and risk of colorectal cancer: an overview from pathophysiology to pharmacological prevention. Front. Pharmacol. 12, 772101. 10.3389/fphar.2021.772101 34744751 PMC8563785

[B100] Lucas C. Barnich N. Nguyen H. T. T. (2017). Microbiota, inflammation and colorectal cancer. Int. J. Mol. Sci. 18 (6), 1310. 10.3390/ijms18061310 28632155 PMC5486131

[B101] Lumeng C. N. Bodzin J. L. Saltiel A. R. (2007). Obesity induces a phenotypic switch in adipose tissue macrophage polarization. J. Clin. Investig. 117 (1), 175–184. 10.1172/JCI29881 17200717 PMC1716210

[B102] Mallet C. Desmeules J. Pegahi R. Eschalier A. (2023). An updated review on the metabolite (AM404)-Mediated central mechanism of action of paracetamol (acetaminophen): experimental evidence and potential clinical impact. J. Pain Res. 16, 1081–1094. 10.2147/JPR.S393809 37016715 PMC10066900

[B103] Mangal N. Erridge S. Habib N. Sadanandam A. Reebye V. Sodergren M. H. (2021). Cannabinoids in the landscape of cancer. J. Cancer Res. Clin. Oncol. 147 (9), 2507–2534. 10.1007/s00432-021-03710-7 34259916 PMC8310855

[B104] Mantovani A. Allavena P. (2015). The interaction of anticancer therapies with tumor-associated macrophages. J. Exp. Med. 212 (4), 435–445. 10.1084/jem.20150295 25753580 PMC4387285

[B105] Martin M. Sun M. Motolani A. Lu T. (2021). The pivotal player: components of NF-κB pathway as promising biomarkers in colorectal cancer. Int. J. Mol. Sci. 22 (14), 7429. 10.3390/ijms22147429 34299049 PMC8303169

[B106] Masjedi A. Hajizadeh F. Beigi Dargani F. Beyzai B. Aksoun M. Hojjat-Farsangi M. (2021). Oncostatin M: a mysterious cytokine in cancers. Int. Immunopharmacol. 90, 107158. 10.1016/j.intimp.2020.107158 33187910

[B107] McCoy A. Araujo-Perez F. Azcarate-Peril A. Yeh J. Sandler R. Keku T. (2013). Fusobacterium is associated with colorectal adenomas. PLoS One 8 (1), e53653. 10.1371/journal.pone.0053653 23335968 PMC3546075

[B108] Meira L. B. Bugni J. M. Green S. L. Lee C. W. Pang B. Borenshtein D. (2008). DNA damage induced by chronic inflammation contributes to colon carcinogenesis in mice. J Clin. Investig. 118 (7), 2516–2525. 10.1172/JCI35073 18521188 PMC2423313

[B109] Michels N. van Aart C. Morisse J. Mullee A. Huybrechts I. (2021). Chronic inflammation towards cancer incidence: a systematic review and meta-analysis of epidemiological studies. Crit. Rev. Oncol. Hematol. 157, 103177. 10.1016/j.critrevonc.2020.103177 33264718

[B110] Milanovic M. Fan D. Belenki D. Däbritz J. Zhao Z. Yu Y. (2018). Senescence-associated reprogramming promotes cancer stemness. Nature 553 (7686), 96–100. 10.1038/nature25167 29258294

[B111] Moossavi S. Bishehsari F. (2012). Inflammation in sporadic colorectal cancer. Arch. Iran. Med. 15 (3), 166–170. 22369306

[B112] Morales-Valencia J. David G. (2021). The contribution of physiological and accelerated aging to cancer progression through senescence-induced inflammation. Front. Oncol. 11, 747822. 10.3389/fonc.2021.747822 34621683 PMC8490756

[B113] Murata M. (2018). Inflammation and cancer. Inflamm. cancer. Environ Health Prev Med. 23 (1), 50. 10.1186/s12199-018-0740-1 PMC6195709 30340457

[B114] Nagy J. A. Benjamin L. Zeng H. Dvorak A. M. Dvorak H. F. (2008). Vascular permeability, vascular hyperpermeability and angiogenesis. Angiogenesis 11 (2), 109–119. 10.1007/s10456-008-9099-z 18293091 PMC2480489

[B115] Nakanishi Y. Nakatsuji M. Seno H. Ishizu S. Akitake-Kawano R. Kanda K. (2011). COX-2 inhibition alters the phenotype of tumor-associated macrophages from M2 to M1 in ApcMin/+ mouse polyps. Carcinogenesis 32 (9), 1333–1339. 10.1093/carcin/bgr128 21730361

[B116] National Academies of Sciences (2017) Engineering, and medicine; health and medicine division; board on population health and public health practice; committee on the health effects of marijuana: an evidence review and research agenda. The health effects of cannabis and cannabinoids: the current state of evidence and recommendations for research. Washington (DC): National Academies Press. 28182367

[B117] Naugler W. E. Sakurai T. Kim S. Maeda S. Kim K. H. Elsharkawy A. M. (2007). Gender disparity in liver cancer due to sex differences in MyD88-dependent IL-6 production. Science 317 (5834), 121–124. 10.1126/science.1140485 17615358

[B118] Nelson K. M. Dahlin J. L. Bisson J. Graham J. Pauli G. F. Walters M. A. (2017). The essential medicinal chemistry of curcumin. J. Med. Chem. 60 (5), 1620–1637. 10.1021/acs.jmedchem.6b00975 28074653 PMC5346970

[bib199] Ng S.-K. Chung D.-J. Chang L.-C. Luo C.-K. Jwo S.-H. Lee Y.-H. (2023). The protective effect of cannabinoids against colorectal cancer cachexia through modulation of inflammation and immune responses. Biomed. Pharmacother. 161, 114467. 10.1016/j.biopha.2023.114467 36871538

[B119] Nolen B. Marks J. Ta’san S. Rand A. Luong T. Wang Y. (2008). Serum biomarker profiles and response to neoadjuvant chemotherapy for locally advanced breast cancer. Breast Cancer Res. 10 (3), R45. 10.1186/bcr2096 18474099 PMC2481492

[B120] Pacher P. Kogan N. Mechoulam R. (2020). Beyond THC and endocannabinoids. Annu. Rev. Pharmacol. Toxicol. 60, 637–659. 10.1146/annurev-pharmtox-010818-021441 31580774

[B121] Pagano C. Savarese B. Coppola L. Navarra G. Avilia G. Laezza C. (2023). Cannabinoids in the modulation of oxidative signaling. Int. J. Mol. Sci. 24 (3), 2513. 10.3390/ijms24032513 36768835 PMC9916673

[B122] Park S. W. Hah J. H. Oh S. M. Jeong W. J. Sung M. W. (2016). 5-lipoxygenase mediates docosahexaenoyl ethanolamide and N-arachidonoyl-L-alanine-induced reactive oxygen species production and inhibition of proliferation of head and neck squamous cell carcinoma cells. BMC Cancer 16, 458. 10.1186/s12885-016-2499-3 27411387 PMC4942960

[B123] Patel M. Horgan P. McMillan D. Edwards J. (2018). NF-κB pathways in the development and progression of colorectal cancer. Transl. Res. 197, 43–56. 10.1016/j.trsl.2018.02.002 29550444

[B124] Patsos H. Hicks D. Dobson R. Greenhough A. Woodman N. Lane J. D. (2005). The endogenous cannabinoid, anandamide, induces cell death in colorectal carcinoma cells: a possible role for cyclooxygenase 2. Gut 54 (12), 1741–1750. 10.1136/gut.2005.073403 16099783 PMC1774787

[B125] Paul D. (2020). The systemic hallmarks of cancer. J. Cancer. Metastasis. Treat. 6, 29. 10.20517/2394-4722.2020.63

[B126] Pellati F. Borgonetti V. Brighenti V. Biagi M. Benvenuti S. Corsi L. (2018). Cannabis sativa L. And nonpsychoactive cannabinoids: their chemistry and role against oxidative stress, inflammation, and cancer. Biomed. Res. Int. 2018, 1691428. 10.1155/2018/1691428 30627539 PMC6304621

[B127] Pennant N. M. Hinton C. V. (2023). The evolution of cannabinoid receptors in cancer. WIREs Mech. Dis. 15 (4), e1602. 10.1002/wsbm.1602 36750231 PMC10484301

[B128] Pikarsky E. Porat R. M. Stein I. Abramovitch R. Amit S. Kasem S. (2004). NF-kappaB functions as a tumour promoter in inflammation-associated cancer. Nature 431 (7007), 461–466. 10.1038/nature02924 15329734

[B129] Pisanti S. Picardi P. D'Alessandro A. Laezza C. Bifulco M. (2013). The endocannabinoid signaling system in cancer. Trends Pharmacol. Sci. 34 (5), 273–282. 10.1016/j.tips.2013.03.003 23602129

[B130] Popivanova B. K. Kitamura K. Wu Y. Kondo T. Kagaya T. Kaneko S. (2008). Blocking TNF-alpha in mice reduces colorectal carcinogenesis associated with chronic colitis. J. Clin. Investig. 118 (2), 560–570. 10.1172/JCI32453 18219394 PMC2213370

[B131] Price J. G. Idoyaga J. Salmon H. Hogstad B. Bigarella C. L. Ghaffari S. (2015). CDKN1A regulates Langerhans cell survival and promotes Treg cell generation upon exposure to ionizing irradiation. Nat. Immunol. 16 (10), 1060–1068. 10.1038/ni.3270 26343536 PMC4620552

[B132] Qin J. Li R. Raes J. Arumugam M. Burgdorf K. S. Manichanh C. (2010). A human gut microbial gene catalogue established by metagenomic sequencing. Nature 464 (7285), 59–65. 10.1038/nature08821 20203603 PMC3779803

[B133] Rao V. Poutahidis T. Ge Z. Nambiar P. Boussahmain C. Wang Y. (2006). Innate immune inflammatory response against enteric bacteria Helicobacter hepaticus induces mammary adenocarcinoma in mice. Cancer Res. 66 (15), 7395–7400. 10.1158/0008-5472.CAN-06-0558 16885333

[B134] Rockwell C. Raman P. Kaplan B. Kaminski N. (2008). A COX-2 metabolite of the endogenous cannabinoid, 2- arachidonyl glycerol, mediates suppression of IL-2 secretion in activated Jurkat T cells. Biochem. Pharmacol. 76 (3), 353–361. 10.1016/j.bcp.2008.05.005 18571623

[B135] Roe K. (2021a). An inflammation classification system using cytokine parameters. Scand. J Immunol 93 (2), e12970. 10.1111/sji.12970 32892387

[B136] Roe K. (2021b). An inflammation classification system using cytokine parameters. Scand. J Immunol 93 (2), e12970. 10.1111/sji.12970 32892387

[B137] Romano M. R. Lograno M. D. (2007). Evidence for the involvement of cannabinoid CB1 receptors in the bimatoprost-induced contractions on the human isolated ciliary muscle. Investig. Ophthalmol. Vis. Sci. 48 (8), 3677–3682. 10.1167/iovs.06-0896 17652738

[B138] Rothwell P. M. Wilson M. Price J. F. Belch J. F. F. Meade T. W. Mehta Z. (2012). Effect of daily aspirin on risk of cancer metastasis: a study of incident cancers during randomised controlled trials. Lancet 379 (9826), 1591–1601. 10.1016/S0140-6736(12)60209-8 22440947

[B139] Rouet-Benzineb P. Aparicio T. Guilmeau S. Pouzet C. Descatoire V. Buyse M. (2004). Leptin counteracts sodium butyrate-induced apoptosis in human colon cancer HT-29 cells via NF-kappaB signaling. J. Biol. Chem. 279 (16), 16495–16502. 10.1074/jbc.M312999200 14752104

[B140] Sahai E. Astsaturov I. Cukierman E. DeNardo D. G. Egeblad M. Evans R. M. (2020). A framework for advancing our understanding of cancer-associated fibroblasts. Nat. Rev. Cancer 20 (3), 174–186. 10.1038/s41568-019-0238-1 31980749 PMC7046529

[B141] Salazar M. Carracedo A. Salanueva I. J. Hernández-Tiedra S. Lorente M. Egia A. (2009). Cannabinoid action induces autophagy-mediated cell death through stimulation of ER stress in human glioma cells. J. Clin. Investig. 119 (5), 1359–1372. 10.1172/jci37948 19425170 PMC2673842

[B142] Saleh T. Tyutyunyk-Massey L. Murray G. F. Alotaibi M. R. Kawale A. S. Elsayed Z. (2019). Tumor cell escape from therapy-induced senescence. Biochem. Pharmacol. 162, 202–212. 10.1016/j.bcp.2018.12.013 30576620

[B143] Schmitt M. Greten F. R. (2021). The inflammatory pathogenesis of colorectal cancer. Nat. Rev. Immunol. 21 (10), 653–667. 10.1038/s41577-021-00534-x 33911231

[B144] Schwitalla S. Ziegler P. K. Horst D. Becker V. Kerle I. Begus-Nahrmann Y. (2013). Loss of p53 in enterocytes generates an inflammatory microenvironment enabling invasion and lymph node metastasis of carcinogen-induced colorectal tumors. Cancer Cell. 23 (1), 93–106. 10.1016/j.ccr.2012.11.014 23273920

[B145] Sethi G. Shanmugam M. K. Ramachandran L. Kumar A. P. Tergaonkar V. (2012). Multifaceted link between cancer and inflammation. Biosci. Rep. 32 (1), 1–15. 10.1042/BSR20100136 21981137

[B146] Shalapour S. Font-Burgada J. di Caro G. Zhong Z. Sanchez-Lopez E. Dhar D. (2015). Immunosuppressive plasma cells impede T-cell-dependent immunogenic chemotherapy. Nature 521 (7550), 94–98. 10.1038/nature14395 25924065 PMC4501632

[B147] Shen X. Rawls J. Randall T. Burcal L. Mpande C. N. Jenkins N. (2010). Molecular characterization of mucosal adherent bacteria and associations with colorectal adenomas. Gut Mic. 1 (3), 138–147. 10.4161/gmic.1.3.12360 PMC2927011 20740058

[B148] Shishodia S. Potdar P. Gairola C. G. Aggarwal B. B. (2003). Curcumin (diferuloylmethane) down-regulates cigarette smoke-induced NF-kappaB activation through inhibition of IkappaBalpha kinase in human lung epithelial cells: correlation with suppression of COX-2, MMP-9 and cyclin D1. Carcinogenesis 24 (7), 1269–1279. 10.1093/carcin/bgg078¡ 12807725

[B149] Silva-Reis R. Silva A. M. S. Oliveira P. A. Cardoso S. M. (2023). Antitumor effects of cannabis sativa bioactive compounds on colorectal carcinogenesis. Biomolecules 13 (5), 764. 10.3390/biom13050764 37238634 PMC10216468

[B150] Silvinato A. Floriano I. Bernardo W. M. (1992)2022). Use of cannabidiol in the treatment of epilepsy: lennox-Gastaut syndrome, Dravet syndrome, and tuberous sclerosis complex. Rev. Assoc. Med. Bras. 68 (10), 1345–1357. 10.1590/1806-9282.2022D689 PMC9683917 36417631

[B151] Sivan A. Corrales L. Hubert N. Williams J. Aquino-Michaels K. Earley Z. M. (2015). Commensal Bifidobacterium promotes antitumor immunity and facilitates anti–PD-L1 efficacy. Science 350 (6264), 1084–1089. 10.1126/science.aac4255 26541606 PMC4873287

[B152] Soleimani A. Rahmani F. Ferns G. Ryzhikov M. Avan A. Hassanian S. M. (2020). Role of the NF-κB signaling pathway in the pathogenesis of colorectal cancer. Gene 726, 144132. 10.1016/j.gene.2019.144132 31669643

[B153] Solinas M. Massi P. Cinquina V. Valenti M. Bolognini D. Gariboldi M. (2013). Cannabidiol, a non-psychoactive cannabinoid compound, inhibits proliferation and invasion in U87-MG and T98G glioma cells through a multitarget effect. PLoS One 8 (10), e76918. 10.1371/journal.pone.0076918 24204703 PMC3804588

[B154] Stattin P. Palmqvist R. Soderberg S. Biessy C. Ardnor B. Hallmans G. Kaaks R. Olsson T. al (2003). Plasma leptin and colorectal cancer risk: a prospective study in Northern Sweden. Oncol. Rep. 10 (6), 2015–2021. 10.3892/or.10.6.2015 14534736

[B155] Stephenson J. Nutma E. Van der Valk P. Amor S. (2018). Inflammation in CNS neurodegenerative diseases. Immunology 154 (2), 204–219. 10.1111/imm.12922 29513402 PMC5980185

[B156] Stocks T. Lukanova A. Johansson M. Rinaldi S. Palmqvist R. Hallmans G. (2008). Components of the metabolic syndrome and colorectal cancer risk; a prospective study. Int. J. Obes. Lond. 32 (2), 304–314. 10.1038/sj.ijo.0803713 17878894

[B157] Sugimoto M. A. Sousa L. P. Pinho V. Perretti M. Teixeira M. M. (2016). Resolution of inflammation: what controls its onset? Front. Immunol. 7, 160. 10.3389/fimmu.2016.00160 27199985 PMC4845539

[B158] Sugimoto N. Ishibashi H. Nakamura H. Yachie A. Ohno-Shosaku T. (2017). Hypoxia-induced inhibition of the endocannabinoid system in glioblastoma cells. Oncol. Rep. 38, 3702–3708. 10.3892/or.2017.6048 29130103

[B159] Sugimoto N. Ishibashi H. Ueda Y. Nakamura H. Yachie A. Ohno-Shosaku T. (2019). Corticosterone inhibits the expression of cannabinoid receptor-1 and cannabinoid receptor agonist-induced decrease in cell viability in glioblastoma cells. Oncol. Lett. 18 (2), 1557–1563. 10.3892/ol.2019.10456 31423223 PMC6607110

[B160] Sun K. Kusminski C. M. Scherer P. E. (2011). Adipose tissue remodeling and obesity. J. Clin. Investig. 121 (6), 2094–2101. 10.1172/JCI45887 21633177 PMC3104761

[B161] Sun X. Casbas-Hernandez P. Bigelow C. Makowski L. Joseph Jerry D. Smith Schneider S. Troester M. A. al (2012). Normal breast tissue of obese women is enriched for macrophage markers and macrophage-associated gene expression. Breast Cancer Res. Treat. 131 (3), 1003–1012. 10.1007/s10549-011-1789-3 22002519 PMC3640411

[B162] Sung H. Ferlay J. Siegel R. L. Laversanne M. Soerjomataram I. Jemal A. (2021). Global Cancer Statistics 2020: GLOBOCAN estimates of incidence and mortality worldwide for 36 cancers in 185 countries. CA Cancer J. Clin. 71 (3), 209–249. 10.3322/caac.21660 33538338

[B163] Suryavanshi S. Zaiachuk M. Pryimak N. Kovalchuk I. Kovalchuk O. (2022). Cannabinoids alleviate the LPS- induced cytokine storm via attenuating NLRP3 inflammasome signaling and TYK2-mediated STAT3 signaling pathways *in vitro* . Cells 11 (9), 1391. 10.3390/cells11091391 35563697 PMC9103143

[B164] Suryavanshi S. v. Kovalchuk I. Kovalchuk O. (2021). Cannabinoids as key regulators of inflammasome signaling: a current perspective. Front. Immunol 11, 3638613613. 10.3389/fimmu.2020.613613 PMC7876066 33584697

[B165] Taniguchi K. Karin M. (2014a). IL-6 and related cytokines as the critical lynchpins between inflammation and cancer. Semin. Immunol. 26 (1), 54–74. 10.1016/j.smim.2014.01.001 24552665

[B166] Taniguchi K. Karin M. (2014b). IL-6 and related cytokines as the critical lynchpins between inflammation and cancer. Semin. Immunol. 26 (1), 54–74. 10.1016/j.smim.2014.01.001 24552665

[B167] Tchkonia T. Morbeck D. E. Von Zglinicki T. Van Deursen J. Lustgarten J. Scrable H. (2010). Fat tissue, aging, and cellular senescence. Aging Cell. 9 (5), 667–684. 10.1111/j.1474-9726.2010.00608.x9726.2010.00608.x 20701600 PMC2941545

[B168] Terzic J. Grivennikov S. Karin E. Karin M. (2010). Inflammation and colon cancer. Gastroenterology 138 (6), 2101–2114. 10.1053/j.gastro.2010.01.058 20420949

[B169] Thapa D. Kang Y. Park P. H. Noh S. K. Lee Y. R. Han S. S. (2012). Anti-tumor activity of the novel hexahydrocannabinol analog LYR-8 in Human colorectal tumor xenograft is mediated through the inhibition of Akt and hypoxia-inducible factor-1α activation. Biol. Pharm. Bull. 35 (6), 924–932. 10.1248/bpb.35.924 22687485

[B170] Thompson P. A. Khatami M. Baglole C. J. Sun J. Harris S. A. Moon E. Y. (2015). Environmental immune disruptors, inflammation and cancer risk. Carcinogenesis 36 (Suppl. 1), S232–S253. 10.1093/carcin/bgv038 26106141 PMC4492068

[B171] Todoric J. Antonucci L. Karin M. (2016). Targeting inflammation in cancer prevention and therapy. Cancer Prev. Res. (Phila) 9 (12), 895–905. 10.1158/1940-6207.CAPR-16-0209 27913448 PMC5142754

[B172] Tuomisto A. E. Mäkinen M. J. Väyrynen J. P. (2019a). Systemic inflammation in colorectal cancer: underlying factors, effects, and prognostic significance. World J. Gastroenterol. 25 (31), 4383–4404. 10.3748/wjg.v25.i31.4383 31496619 PMC6710177

[B173] Tuomisto A. E. Mäkinen M. J. Väyrynen J. P. (2019b). Systemic inflammation in colorectal cancer: underlying factors, effects, and prognostic significance. World J. Gastroenterol. 25 (31), 4383–4404. 10.3748/wjg.v25.i31.4383 31496619 PMC6710177

[B174] Turkmen K. (2017). Inflammation, oxidative stress, apoptosis, and autophagy in diabetes mellitus and diabetic kidney disease: the Four Horsemen of the Apocalypse. Int. Urol. Nephrol. 49 (5), 837–844. 10.1007/s11255-016-1488-4 28035619

[B175] Uchiyama T. Takahashi H. Sugiyama M. Sakai E. Endo H. Hosono K. (2011). Leptin receptor is involved in STAT3 activation in human colorectal adenoma. Cancer Sci. 102 (2), 367–372. 10.1111/j.1349-7006.2010.01803.x 21166956

[B176] Udoh M. Santiago M. Devenish S. McGregor I. Connor M. (2019). Cannabichromene is a cannabinoid CB2 receptor agonist. Br. J. Pharmacol. 176, 4537–4547. 10.1111/bph.14815 31368508 PMC6932936

[B177] Vara D. Salazar M. Olea-Herrero N. Guzmán M. Velasco G. Díaz-Laviada I. (2011). Anti-tumoral action of cannabinoids on hepatocellular carcinoma: role of AMPK-dependent activation of autophagy. Cell. Death Differ. 18 (7), 1099–1111. 10.1038/cdd.2011.32 21475304 PMC3131949

[B178] Velasco G. Sánchez C. Guzmán M. (2016). Anticancer mechanisms of cannabinoids. Curr. Oncol. 23 (2), S23–S32. 10.3747/co.23.3080 PMC4791144 27022311

[B179] Vétizou M. Pitt J. M. Daillère R. Lepage P. Waldschmitt N. Flament C. (2015). Anticancer immunotherapy by CTLA-4 blockade relies on the gut microbiota. Science 350 (6264), 1079–1084. 10.1126/science.aad1329 26541610 PMC4721659

[B180] Vipperla K. Keefe S. (2016). Diet, microbiota, and dysbiosis: a ‘recipe’ for colorectal cancer. Food Funct. 7 (4), 1731–1740. 10.1039/c5fo01276g 26840037 PMC6501806

[B181] Volkow N. Hampson A. Baler R. (2017). Don’t worry, be happy: endocannabinoids and cannabis at the intersection of stress and reward. Annu. Rev. Pharmacol. Toxicol. 57, 285–308. 10.1146/annurev-pharmtox-010716-104615 27618739

[B182] Wamsteeker J. I. Kuzmiski J. B. Bains J. S. (2010). Repeated stress impairs endocannabinoid signaling in the paraventricular nucleus of the hypothalamus. J. Neurosci. 30, 11188–11196. 10.1523/JNEUROSCI.1046-10.2010 20720126 PMC6633493

[B183] Wang D. Chen J. Chen H. Duan Z. Xu Q. Wei M. Wang L. Zhong M. al (2012). Leptin regulates proliferation and apoptosis of colorectal carcinoma through PI3K/Akt/mTOR signalling pathway. J. Biosci. 37 (1), 91–101. 10.1007/s12038-011-9172-4 22357207

[B184] Wang D. Dubois R. (2010). The role of COX-2 in intestinal inflammation and colorectal cancer. Oncogene 29 (6), 781–788. 10.1038/onc.2009.421 19946329 PMC3181054

[B185] Wang H. Li M. Rinehart J. J. Zhang R. (2004). Pretreatment with dexamethasone increases antitumor activity of carboplatin and gemcitabine in mice bearing human cancer xenografts: *in vivo* activity, pharmacokinetics, and clinical implications for cancer chemotherapy. Clin. Cancer Res. 10 (5), 1633–1644. 10.1158/1078-0432.ccr-0829-3 15014014

[B186] Weber A. Ni J. Ling K. H. Acheampong A. Tang-Liu D. D. Burk R. (2004). Formation of prostamides from anandamide in FAAH knockout mice analyzed by HPLC with tandem mass spectrometry. J. Lipid Res. 45 (4), 757–763. 10.1194/jlr.M300475-JLR200 14729864

[B187] Weisberg S. P. McCann D. Desai M. Rosenbaum M. Leibel R. L. Ferrante A. W. (2003). Obesity is associated with macrophage accumulation in adipose tissue. J. Clin. Investig. 112 (12), 1796–1808. 10.1172/JCI19246 14679176 PMC296995

[B188] West N. Powrie F. (2015). Immunotherapy not working? Check your microbiota. Cancer Cell. 28 (6), 687–689. 10.1016/j.ccell.2015.11.010 26678336

[B189] Westendorf A. M. Skibbe K. Adamczyk A. Buer J. Geffers R. Hansen W. (2017). Hypoxia enhances immunosuppression by inhibiting CD4+ effector T cell function and promoting Treg activity. Cell. Physiol. Biochem. 41 (4), 1271–1284. 10.1159/000464429 28278498

[B190] Wong R. S. Y. (2019). Role of nonsteroidal anti-inflammatory drugs (NSAIDs) in cancer prevention and cancer promotion. Adv. Pharmacol. Sci. 2019, 3418975. 10.1155/2019/3418975 30838040 PMC6374867

[B191] Woodward D. F. Liang Y. Krauss A. H. (2008). Prostamides (prostaglandin-ethanolamides) and their pharmacology. Br. J. Pharmacol. 153 (3), 410–419. 10.1038/sj.bjp.0707434 17721551 PMC2241799

[B192] Xiao H. Yang C. S. (2008). Combination regimen with statins and NSAIDs: a promising strategy for cancer chemoprevention. Int. J. Cancer 123 (5), 983–990. 10.1002/ijc.23718 18548583

[B193] Xu J. Ye Y. Zhang H. Szmitkowski M. Mäkinen M. Li P. (2016). Diagnostic and prognostic value of serum interleukin-6 in colorectal cancer. Med. Baltim. 95 (2), e2502. 10.1097/MD.0000000000002502 PMC4718291 26765465

[B194] Xu M. Wang Y. Xia R. Wei Y. Wei X. (2021). Role of the CCL2-CCR2 signalling axis in cancer: mechanisms and therapeutic targeting. Cell. Prolif. 54 (10), e13115. 10.1111/cpr.13115 34464477 PMC8488570

[B195] Yarla N. S. Bishayee A. Sethi G. Reddanna P. Kalle A. M. Dhananjaya B. L. (2016). Targeting arachidonic acid pathway by natural products for cancer prevention and therapy. Seminars Cancer Biol. 40–41, 48–81. 10.1016/j.semcancer.2016.02.001 26853158

[B196] Yu H. Pardoll D. Jove R. (2009). STATs in cancer inflammation and immunity: a leading role for STAT3. Nat. Rev. Cancer 9 (11), 798–809. 10.1038/nrc2734 19851315 PMC4856025

[B197] Yu Y. Fang J. (2015). Gut microbiota and colorectal cancer. Gastro Tumors 2 (1), 26–32. 10.1159/000380892 PMC4668798 26674881

[B198] Zappavigna S. Cossu A. M. Grimaldi A. Bocchetti M. Ferraro G. A. Nicoletti G. F. (2020). Anti-inflammatory drugs as anticancer agents. I J Mol Sci. 21 (7), 2605. 10.3390/ijms21072605 PMC7177823 32283655

